# Stromal senescence establishes an immunosuppressive microenvironment that drives tumorigenesis

**DOI:** 10.1038/ncomms11762

**Published:** 2016-06-08

**Authors:** Megan K. Ruhland, Andrew J. Loza, Aude-Helene Capietto, Xianmin Luo, Brett L. Knolhoff, Kevin C. Flanagan, Brian A. Belt, Elise Alspach, Kathleen Leahy, Jingqin Luo, Andras Schaffer, John R. Edwards, Gregory Longmore, Roberta Faccio, David G. DeNardo, Sheila A. Stewart

**Affiliations:** 1 Department of Cell Biology and Physiology, Washington University School of Medicine, 660 South Euclid Avenue, St. Louis, Missouri 63110, USA; 2 Department of Medicine,Washington University School of Medicine, 660 South Euclid Avenue, St. Louis, Missouri 63110, USA; 3 Department of Orthopedic Surgery, Washington University School of Medicine, 660 South Euclid Avenue, St. Louis, Missouri 63110, USA; 4 Department of Surgery, Washington University School of Medicine, 660 South Euclid Avenue, St. Louis, Missouri 63110, USA; 5 Department of Pathology and Immunology, Washington University School of Medicine, 660 South Euclid Avenue, St. Louis, Missouri 63110, USA; 6 Division of Biostatistics, Washington University School of Medicine, 660 South Euclid Avenue, St. Louis, Missouri 63110, USA; 7 Siteman Cancer Center, Washington University School of Medicine, 660 South Euclid Avenue, St. Louis, Missouri 63110, USA; 8 Center For Pharmacogenomics, Washington University School of Medicine, 660 South Euclid Avenue, St. Louis, Missouri 63110, USA; 9 ICCE Institute, Washington University School of Medicine, 660 South Euclid Avenue, St Louis, Missouri 63110, USA

## Abstract

Age is a significant risk factor for the development of cancer. However, the mechanisms that drive age-related increases in cancer remain poorly understood. To determine if senescent stromal cells influence tumorigenesis, we develop a mouse model that mimics the aged skin microenvironment. Using this model, here we find that senescent stromal cells are sufficient to drive localized increases in suppressive myeloid cells that contributed to tumour promotion. Further, we find that the stromal-derived senescence-associated secretory phenotype factor interleukin-6 orchestrates both increases in suppressive myeloid cells and their ability to inhibit anti-tumour T-cell responses. Significantly, in aged, cancer-free individuals, we find similar increases in immune cells that also localize near senescent stromal cells. This work provides evidence that the accumulation of senescent stromal cells is sufficient to establish a tumour-permissive, chronic inflammatory microenvironment that can shelter incipient tumour cells, thus allowing them to proliferate and progress unabated by the immune system.

## Abstract (web summary)

The risk of developing cancer increases with age. Here, the authors address the contribution of age-dependent accumulation of senescent cells within the tumour stroma compartment and show that senescent cells increase the infiltration of myeloid-derived suppressor cells that inhibit cytotoxic T-cells, thus facilitating tumour outgrowth.

## Graphical abstract

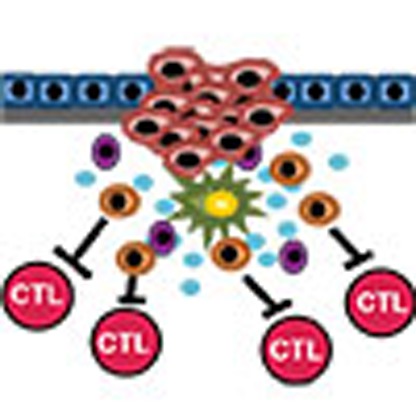

Age significantly affects an individual's risk for developing cancer^1^. The factors that contribute to age-related increases in cancer are thought to include accumulation of stochastic mutations within incipient tumour cells and collaborative stromal changes that together drive tumorigenesis. While a plethora of cell-autonomous mutations have been shown to contribute to cellular transformation, how an aging stromal compartment develops and supports tumour outgrowth remains poorly understood. Inflammation may provide a link that explains how changes in the stromal compartment contribute to age-related increases in tumour development. Indeed, older individuals experience systemic changes in mediators of chronic inflammation including increases in cytokines and various immune cells such as immunosuppressive myeloid cells^2^,^3^,^4^,^5^,^6^. It remains unclear what drives these increases, but one contributing factor may be the accumulation of senescent cells that is known to occur with age^7^,^8^,^9^. Supporting the putative role of senescent cells in age-related increases in tumorigenesis is recent work showing that depletion of senescent cells in mice leads to a significant reduction in tumorigenesis^10^. However, the mechanisms that underlie this reduction remain to be addressed.

Senescent cells are metabolically active cells that are characterized by an irreversible growth arrest. In addition, senescent cells express the cell cycle inhibitor p16^INK4A^ (p16), senescence-associated β-galatosidase (SA-βgal), and an altered expression profile known as the senescence-associated secretory phenotype (SASP)^11^. Among the SASP cytokines, interleukin-6 (IL-6) is considered a canonical inflammatory factor^12^. IL-6 is elevated with age and coincides with increases in both circulating immunosuppressive myeloid cells and cancer incidence^2^,^6^. The possibility that stromal-derived SASP factors, including IL-6, mediate the establishment of chronic inflammation that predisposes a tissue to tumour outgrowth is intriguing.

Senescence plays a paradoxical role in tumorigenesis, being both tumour-promoting and tumour-suppressive depending on the cell in which senescence occurs. Indeed, in some tumour models, senescent neoplastic cells can stimulate immune-mediated tumour cell clearance and thus, in this context, senescence functions as a potent tumour-suppressive mechanism^13^. However, in immune-compromised settings, when admixed with tumour cells, senescent stromal cells actively promote tumour growth through paracrine mechanisms^14^,^15^,^16^,^17^,^18^,^19^. These findings raise two important questions in the setting of an active immune system; (1) how do incipient tumour cells that arise within a senescent stromal compartment evade immune clearance and (2) can senescence within the stromal compartment affect the host immune response and adopt a pro-tumorigenic role? To address these important questions, we created an immune-competent mouse model to interrogate the role senescent stromal cells play in the preneoplastic, inflammatory microenvironment. Upon inducing senescence in the mesenchymal compartment, we find that in the absence of existing tumour cells, senescent stromal cells are sufficient to create an immunosuppressed environment, reminiscent of what we find in aging human skin. Further, we find that senescence-established immunosuppression facilitated tumour outgrowth by increasing myeloid-derived suppressor cells (MDSCs) capable of inhibiting CD8^+^ T-cell function. Together, these findings suggest a mechanism whereby senescent stromal cells contribute to age-related increases in tumorigenesis through the creation of local regions of immunosuppression.

## Results

### Senescent stromal cells drive increased inflammation

To determine if stromal-derived SASP affects the immune microenvironment, we developed a genetically engineered mouse to spatially and temporally control senescence activation exclusively in the stromal compartment^20^. Mice bearing a stromal-specific, tamoxifen (TAM)-inducible Cre-recombinase under the control of the pro-alpha 2(I)collagen promoter^21^ were mated to mice that conditionally activate expression of the cell cycle inhibitor *p27*^*Kip1*^ from the ROSA26 locus (ROSAlox-stop-lox-*p27*^*Kip1*^IRESGFP) (refs 21, 22). The *p27*^*Kip1*^ allele was used because it robustly activates senescence and SASP expression reminiscent of cells induced to senescence through telomere dysfunction, DNA damage-induced senescence and oncogene-induced senescence^23^. To first verify the relevance of p27^Kip1^ in age-related senescence, we stained human skin samples and found age-dependent increases in stromal p27^Kip1^ expression (Supplementary Fig. 1a,b). Thus p27^Kip1^ is a valid model for interrogating senescence-associated phenotypes.

**Figure 1:**
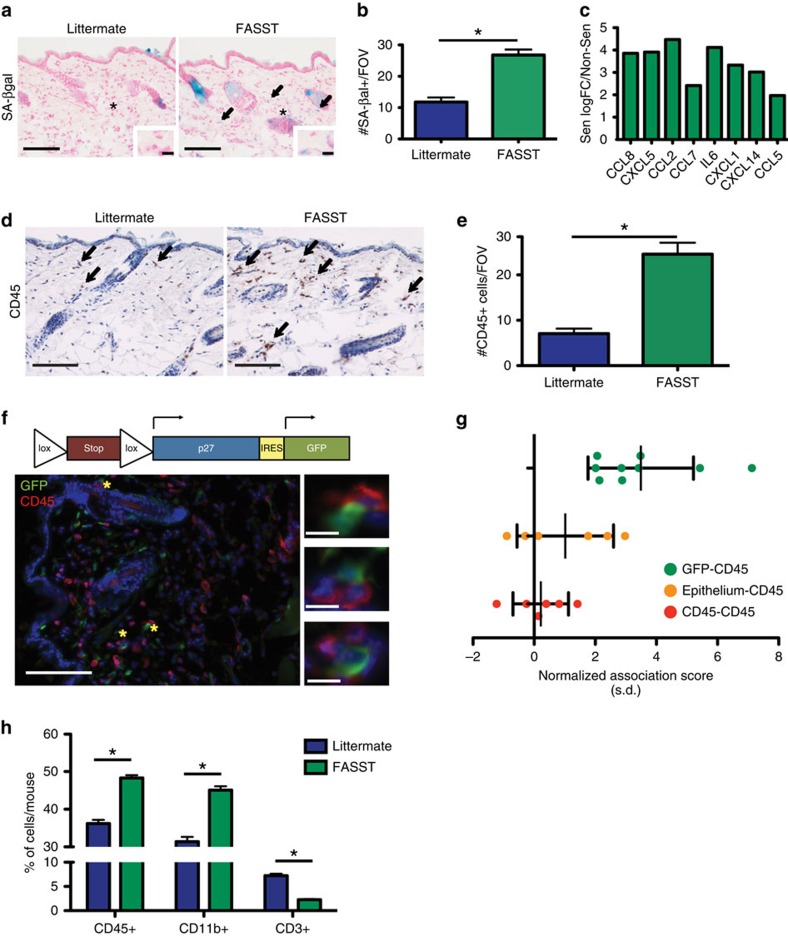
Senescent stromal cells induce local inflammation. (**a**) Senescence activation in FASST or littermate mice. Senescence-associated β-galactosidase (SA-βgal) staining (blue) of skin (arrows). Scale bar, 100 or 10 μm in zoomed images. * site of zoomed image. Representative images. (**b**) Quantification of SA-βgal^+^ cells per × 20 field of view (FOV). *P-value<0.05 by Student's *t*-test. Error bars represent s.e.m. Representative experiment. *n*=10. (**c**) RNA-seq shows expression of multiple cytokines and chemokines significantly increased in senescent MSFs (FDR<0.05, edgeR). Graph is log fold change (logFC) expression in Sen compared with Non-Sen MSFs. *n*=3. (**d**) CD45 IHC staining of skin from FASST or littermate mice. Arrows indicate CD45^+^ cells. Scale bar, 100 μm. Representative images. (**e**) Quantification of CD45^+^ cells per × 20 FOV. *P value<0.05 by Student's *t*-test. Error bars represent s.e.m. Representative experiment. *n*=9. (**f**) Schematic of transgenic expression cassette (top). Below, IF co-staining for transgenic GFP expression (green) and CD45^+^ cells (red). Nuclei stained with DAPI (blue). Scale bar, 100 or 10 μm in zoomed image. * site of zoomed images (right). Representative image. (**g**) Spatial analysis of CD45^+^ leukocytes and GFP^+^ stromal cells. Data show median distance between CD45^+^ leukocytes and GFP^+^ stromal cells (green) is significantly smaller than chance (mean normalized association score 3.5, 95% confidence interval (CI) 2.4–4.6). In the absence of senescent cells (littermate control), the observed distance distribution of immune cells to the epithelial layer (orange) is not significantly different from random (Epithelium-CD45) (mean normalized association score=1.0, 95% CI −0.2 to 2.2). Immune cell-immune cell (CD45-CD45) distances (red) are not significantly different from random (mean association statistic=0.2, 95% CI −0.5 to 0.9). Error bars represent s.d. *n*=6–9. (**h**) Flow cytometry analysis of FASST and littermate control skin. Data represents CD45^+^, CD45^+^CD11b^+^ or CD45^+^CD3^+^ populations as a percentage of live cells per mouse skin. *P value <0.05 by Student's *t*-test. Data are presented as mean %+s.e.m. shown as error bars. *n*=3. FDR, false discovery rate.

To establish the affect of stromal senescence on tumorigenesis, we first characterized our mouse model, termed the FASST mouse (fibroblasts accelerate stromal supported tumorigenesis), which upon TAM administration activates the *p27*^*Kip1*^ allele within cells of mesenchymal origin. Tissue samples were obtained from 6-week-old FASST and littermate control mice 2 weeks after transgene activation with TAM. To identify senescent cells, skin sections were stained for the senescence marker, senescence-associated β-galactosidase (SA-βgal) (Fig. 1a)^7^. We observed a significant increase in the number of senescent cells within the skin stroma of FASST mice compared with littermate controls (Fig. 1a,b) as well as within the mammary gland, lung (data not shown) and bone^20^, reflecting the mesenchymal nature of the collagen promoter used in our system. Importantly, this finding mimicked the mosaic pattern of senescent cell accumulation observed in aged human tissues^7^.

Senescent stromal cells display an altered, pro-tumorigenic gene expression profile that is characterized by the increased expression of numerous mitogens, extracellular matrix remodelling enzymes, cytokines and chemokines^14^,^15^,^16^,^17^,^18^,^19^. To determine if senescent stromal cells from FASST mice displayed a similar phenotype, we next performed RNA sequencing (RNA-Seq) analysis on TAM treated mouse skin fibroblasts (MSFs) (Fig. 1c). Our analysis demonstrated that mitogens (including PDGFα and IGF2), extracellular matrix remodelling enzymes (including MMP3), and numerous cytokines and chemokines were increased in senescent versus non-senescent MSFs (data not shown). Gene set enrichment analysis (GSEA) revealed a specific enrichment of factors in the JAK-STAT pathway known to modulate immune cells (*P*<10^−3^, GSEA)^24^,^25^. Leading-edge analysis suggested multiple genes contributed to the core enrichment of the pathway (Supplementary Fig. 2a). Compared with non-senescent MSFs, senescent MSFs more robustly expressed the canonical SASP factor IL-6 as well as other inflammatory mediators including CXCL5, CXCL1, CCL2 and CCL5 (Fig. 1c). Upregulation of factors was validated using quantitative reverse transcription PCR (qRT-PCR) (Supplementary Fig. 2b).

Given the increased expression of inflammatory cytokines detected in senescent versus non-senescent MSFs, we next asked if these cells played a role in regulating local inflammation. Using an antibody against CD45, a pan-leukocyte marker, we found 3.5 times as many immune cells in the skin of FASST mice than in littermate controls (Fig. 1d,e), suggesting senescent stromal cells promote local inflammation. In addition, the increased CD45^+^ cells appeared to preferentially localize around senescent fibroblasts. To directly measure this association, we used a spatial analysis technique to examine the relative localization of GFP^+^ senescent cells (green) and CD45^+^ immune cells (red) in co-stained FASST skin (Fig. 1f). An association score for each sample was computed by taking the difference between the observed and random chance nearest-neighbour distances normalized by the s.d. that was determined by random sampling. Positive values indicate association of the two cell types, values of zero indicate the distance distribution matches what is expected by chance, and negative values indicate repulsion. The observed median distance from each immune cell to the nearest senescent fibroblast was found to be significantly smaller than chance would predict in all samples (values>0) (Fig. 1g). Spatial analysis of littermate control skin showed that other potential influences on localization including a bias towards the epithelial layer (orange) or immune cell to immune cell preferential interactions (red) were not present (Fig. 1g). This increase in immune cell number, and localization bias towards senescent cells within FASST skin, strongly suggested that senescent stromal-derived SASP orchestrates a localized inflammatory environment.

Increases in inflammation and a notable shift in immune cell localization led us to next perform polychromatic flow cytometry staining to identify the cell types present in the senescent microenvironment. We stained cells derived from the skin of FASST and littermate mice with immune cell markers and performed flow analysis. Similar to our immunohistochemistry (IHC) analyses, flow cytometric profiling revealed a significant increase in CD45^+^ cells in the FASST skin compared with littermate controls (Fig. 1h). Further analysis revealed that many of these cells were CD11b^+^ myeloid cells. We also found that CD45^+^CD3^+^ lymphocytes were markedly decreased in FASST skin (Fig. 1h). Increases in myeloid cells and concurrent decreases in lymphocytes can be indicative of an immunosuppressive environment.

### Senescent stroma increases CD11b^+^Ly6G^Hi^ and T_reg_ cells

**Figure 2:**
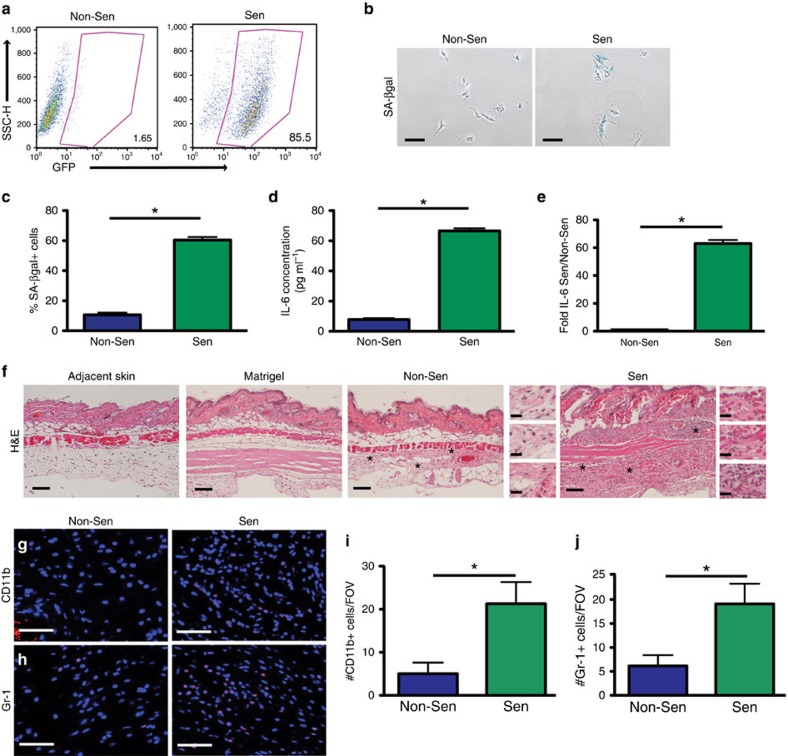
Senescent fibroblasts drive inflammation. (**a**) MSFs isolated from FASST mice show activation of the GFP transgene following addition of 10 μM TAM. Flow cytometry plots with the percent of cells expressing GFP (lower right corner) in non-senescent (Non-Sen) and senescent (Sen) MSFs. Representative plot. *n*=5. (**b**) Senescence-associated β-galactosidase (SA-βgal) staining (blue) on MSFs from FASST mice treated with 10 μM TAM (Sen) or vehicle (Non-Sen). Scale bar, 100 μm. Representative images. (**c**) Quantification of SA-βgal staining. Data are presented as mean percent positive cells+s.e.m. * indicates *P* value <0.05 by Student's *t*-test. *n*=3. (**d**) Quantification of secreted IL-6 protein from Sen or Non-Sen MSFs. Data represents the mean concentration of IL-6 in pg ml^−1^+s.e.m. from medium conditioned for 24 h. Protein concentration was subsequently measured using ELISA. *P value <0.05 by Student's *t*-test. *n*=3. (**e**) IL-6 mRNA expression from MSFs (Sen or Non-Sen) measured by qRT–PCR. Graph represents mean IL-6 mRNA expression in senescent versus non-senescent MSFs. * indicates *P* value<0.05 by Student's *t*-test. Error bars are s.e.m. Representative experiment. *n*=10. (**f**) H&E staining of adjacent skin, matrigel alone, non-senescent (Non-Sen) and senescent (Sen) subcutaneous isografts. Scale bar, 100 μm. Representative images. * indicates site of zoomed images (right). Scale bar, 20 μm in the zoomed images. *n*=4–6. (**g**) IF for CD11b^+^ (red) cells present in Non-Sen and Sen subcutaneous isografts. Scale bar, 100 μm. Representative images. *n*=3. (**h**) IF for Gr-1^+^ (red) cells present in Non-Sen and Sen subcutaneous isografts. Scale bar, 100 μM. Representative images. *n*=3. (**i**) Quantification of CD11b^+^ cells shown in **g**. *indicates *P* value <0.05 by Student's *t*-test. Data are presented as the mean+s.e.m. of CD11b^+^ cells per × 20 field of view (FOV). Representative experiment. *n*=3. (**j**) Quantification of Gr-1^+^ cells shown in **h**. * indicates *P* value <0.05 by Student's *t*-test. Data are presented as the mean+s.e.m. of Gr-1^+^ cells per × 20 FOV. Representative experiment. *n*=3. H&E, haematoxylin and eosin; qRT–PCR, quantitative PCR with reverse transcription.

To elucidate how senescent stromal cells alter inflammation, we interrogated the identity of the various cell lineages recruited to a senescent microenvironment. To provide a tractable model, we isolated MSFs from FASST mice and induced them to senesce *in vitro* by the addition of TAM. We then used additional markers including MHCII, F4/80, Ly6C, CD11c, CD49b, Ly6G, CD4 and CD8, to identify macrophages, dendritic cells, natural killer (NK) cells, granulocytes and CD4^+^ and CD8^+^ T-cell subsets. As expected, TAM treatment led to the induction of transgene expression (GFP^+^), growth arrest, increases in SA-βgal^+^ cells, increased expression of the canonical SASP-factor IL-6, and elevated expression of fibroblast activation protein (FAP), indicating they are activated stromal cells (Fig. 2a–e and Supplementary Fig. 3a–c). Further, as would be expected by our GSEA analysis (Supplementary Fig. 2a), senescent MSFs displayed increased levels of p-STAT3 (Supplementary Fig. 3d,e). To determine how senescent MSFs influenced the host immune response, we injected senescent or non-senescent MSFs subcutaneously into syngeneic, wild-type mice. Histological analyses revealed that the tissue architecture found at sites where matrigel alone or non-senescent MSFs were injected was similar to the normal adjacent skin (Fig. 2f). This finding indicates that at the time of analysis the injection itself did not elicit an inflammatory response. In contrast, senescent MSFs dramatically increased the number of inflammatory cells and extracellular matrix deposition (Fig. 2f). While senescent tumour cells are reportedly cleared from tissues by the immune system^26^, GFP^+^, IL-6-expressing senescent MSFs persisted within senescent isografts (Fig. 2f and Supplementary Fig. 3f–h), indicating that the senescent MSFs were not cleared by the immune system. Subsequent immunofluorescence (IF) staining revealed that the presence of senescent MSFs led to a 4.3-fold increase in CD11b^+^ myeloid cells (Fig. 2g,i). This finding is consistent with increases seen in the FASST mouse skin (Fig. 1h). Additionally, Gr-1^+^ myeloid cells were significantly elevated by 3.1-fold in senescent compared with non-senescent isografts (Fig. 2h,j). These data suggest that senescent stromal cells increase inflammation characterized by myeloid cell infiltration.

**Figure 3:**
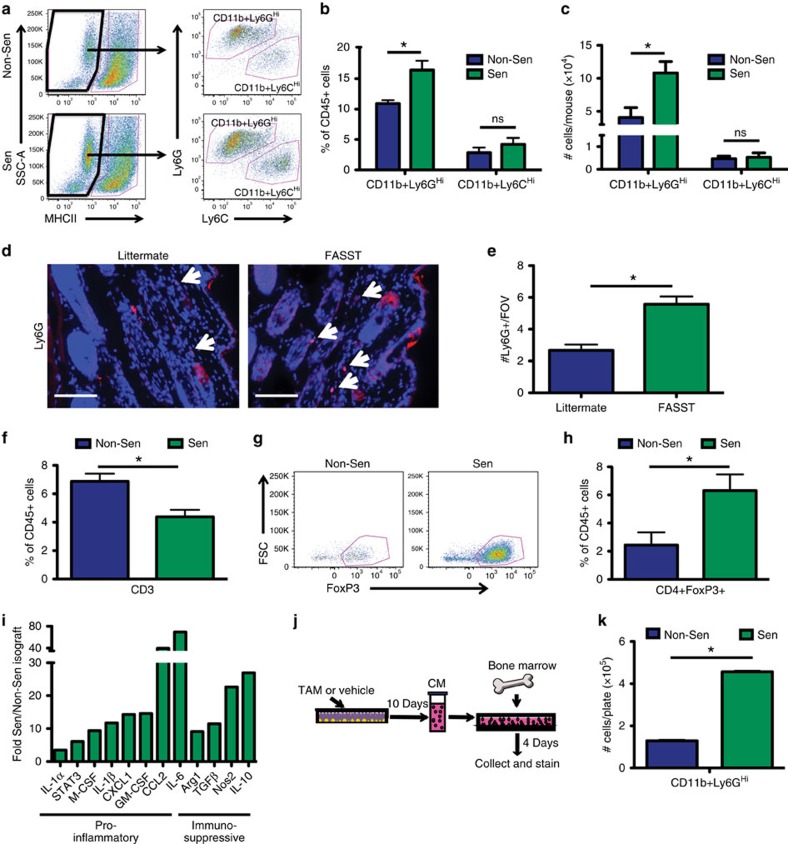
Senescent stromal cells promote the development of an immunosuppressive microenvironment. (**a**) MHCII^Lo^Ly6G^Hi^ and MHCII^Lo^Ly6C^Hi^ gating strategy for non-senescent (Non-Sen) and senescence (Sen) isografts. Cells previously gated as CD45^+^CD11b^+^MHCII^Lo^ (left). MHCII^Lo^ population is further gated for Ly6G and Ly6C (right). (**b**) Quantification of flow cytometry in **a**. Data represents CD11b^+^Ly6G^Hi^ or CD11b^+^Ly6C^Hi^ cells as % of CD45^+^ cells. *P value <0.05 by Student's *t*-test. ns is not significant. Data are mean %+s.e.m. *n*=3. (**c**) Quantification of CD11b^+^Ly6G^Hi^ or CD11b^+^Ly6C^Hi^ cells represented as number of cells per mouse. *P value <0.05 by Student's *t*-test. ns is not significant. Data are mean+s.e.m. *n*=3. (**d**) IF staining for Ly6G^+^ cells (red) in FASST skin. Arrows indicate Ly6G^+^ cells. Scale bar, 100 μM. (**e**) Quantification of Ly6G^+^ cells shown in **d**. *P value <0.05 by Student's *t*-test. Data are mean+s.e.m. *n*=36–37. (**f**) Quantification of CD3^+^ T cells in Sen versus Non-Sen isografts. CD3^+^ cells were previously gated as CD45^+^. *P value<0.05 by Student's *t*-test. Data are mean %+s.e.m. *n*=3–4. (**g**) T regulatory cell (T_reg_) gating for Non-Sen and Sen isografts. Cells previously gated as CD45^+^CD3^+^CD4^+^. (**h**) Quantification of flow cytometry in **g**. *P value <0.05 by Student's *t*-test. Data are mean %+s.e.m. of CD4^+^FoxP3^+^ cells in the CD45^+^ population. *n*=4. (**i**) qRT–PCR analysis of Sen versus Non-Sen isografts shows significant (*P*<0.05, Student's *t*-test) senescence-dependent enrichment of pro-inflammatory and immunosuppressive gene expression *in vivo*. Data are mean fold increase+s.e.m. of the dCT normalized to GAPDH. *n*=11–16. (**j**) Schematic representation of CM assay testing the SASP's impact on naive bone marrow. Non-senescent (vehicle), senescent (TAM). (**k**) Flow cytometry analysis of conditioned (Non-Sen or Sen) medium-treated naive bone marrow cells from set-up in j. *P-value<0.05, Student's *t*-test. Data are mean # of cells per plate+s.e.m. Representative experiment, *n*=4. Data are derived from experiment shown in Fig. 7b thus Sen-CM and Non-Sen-CM contain IgG control antibody. qRT–PCR, quantitative PCR with reverse transcription.

We next used flow cytometry to define the immune cell types present within the senescent microenvironment. We disaggregated isografts containing MSFs and stained the cells with antibodies against immune cell markers (gating strategy Supplementary Fig. 4a). We confirmed that senescent MSFs significantly increased the numbers of CD11b^+^ myeloid cells (data not shown). This is consistent with the staining shown in Fig. 2g and FASST mouse skin staining shown in Fig. 1h. Further, these CD11b^+^ cells expressed low levels of MHCII and high levels of Ly6G (CD11b^+^Ly6G^Hi^) (Fig. 3a,b). No changes were observed in the CD11b^+^MHCII^Lo^Ly6C^Hi^ population (CD11b^+^Ly6C^Hi^) (Fig. 3a–c). Analysis of cell number revealed CD11b^+^Ly6G^Hi^ cells were 2.7-fold greater in number in the presence of senescent compared with non-senescent MSFs (Fig. 3c). Importantly, immunofluorescent staining (IF) staining of FASST skin, where activation of senescence is mosaic in nature, demonstrated a consistent increase in Ly6G^+^ cells (Fig. 3d,e). Analysis of skin from 24-month-old mice also revealed a significant increase in Ly6G^+^ cells (Supplementary Fig. 5a,b) coincident with increases in SA-βgal positive cells within the stromal compartment (Supplementary Fig. 5c,d), suggesting a naturally aging microenvironment drives increases in these cells. Finally, lymphocyte staining of cells obtained from the isografts resembled that found in the FASST mouse skin, showing significant decreases in the percentage of CD3^+^ cells (Fig. 3f, Fig. 1h). However, no significant changes were detected for natural killer (NK) cells, CD4^+^ or CD8^+^ T-cell subsets, or CD4/CD8 T-cell ratio (Supplementary Fig. 5e–h). Using CD11b^+^MHCII^+^F4/80^+^ as a marker for macrophages and CD11b^+^MHCII^+^F4/80-CD11c^+^ to identify dendritic cells, we detected no significant changes in the percentages of these myeloid cells (data not shown). Together, these findings suggest that the isograft system robustly reflects changes within the FASST mouse and naturally aged skin.

CD11b^+^Ly6G^Hi^ and CD11b^+^Ly6C^Hi^ myeloid cells are phenotypically consistent with granulocytic and monocytic MDSCs and are often associated with immunosuppression^27^,^28^. In addition, these myeloid cell subsets have been implicated in the induction of other immunosuppressive cell types including FOXP3^+^ T regulatory cells (T_reg_) (refs 28, 29). We noted a significant 2.6-fold increase in CD3^+^CD4^+^FoxP3^+^ T_reg_ cells in senescent microenvironments (Fig. 3g,h, gating strategy Supplementary Fig. 4b). While no changes in number or percentage of CD4^+^ T cells were detected, the increases in FoxP3^+^ cells within the CD4^+^ T-cell subset is consistent with a functional shift towards an immunosuppressive T-cell population. In agreement with this shift, we found significant increases in immunosuppressive factors within senescent isografts. Indeed, Arg1, Nos2, TGF-β and IL-10 were significantly increased along with other pro-inflammatory cytokines and chemokines including IL-6, CCL2, GM-CSF, M-CSF, IL1-β, IL1-α and CXCL1 in the senescent microenvironment (Fig. 3i). Further, expression of the transcription factor STAT3 that can drive SASP expression as well as immunosuppressive inflammation^30^, was also significantly increased in senescent versus non-senescent isografts (Fig. 3i). Together these data demonstrate that the presence of senescent stromal cells leads to the development of an immunosuppressive microenvironment.

The increased presence of immunosuppressive cell types and signalling molecules in the senescent microenvironment led us to ask if senescence-derived factors were sufficient to drive increases in suppressive myeloid cells. Thus, we generated conditioned medium (CM) from non-senescent or senescent MSFs and placed it on naive bone marrow cells for 96 h and then stained cells with a panel of antibodies against myeloid surface markers before flow cytometric analysis (Fig. 3j). This analysis revealed that CM from senescent MSFs led to a 3.5-fold increase in CD11b^+^Ly6G^Hi^ cells (Fig. 3k), which is the same cell type increased *in vivo* in the presence of senescent MSFs.

**Figure 4:**
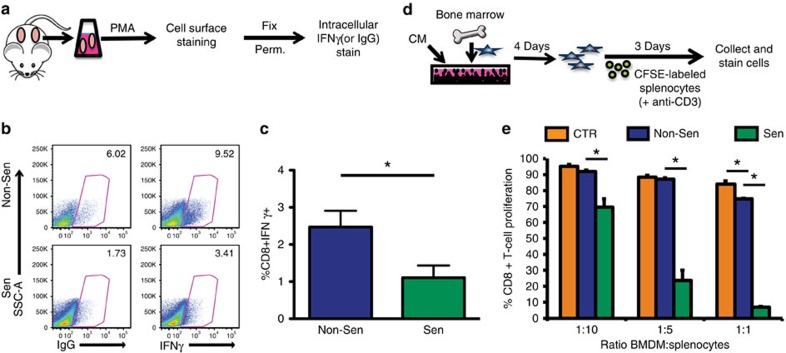
Senescent fibroblasts reduce CD8^+^ T-cell responsiveness. (**a**) Schematic representation of *ex vivo* CD8^+^ T-cell stimulation assay. Cells from isograft digests were stimulated with PMA. Cells were then stained for cell surface markers, fixed, permeabilized (Perm.) and stained for intracellular IFNγ. IgG antibody is used as a control. (**b**) Flow cytometry following *ex vivo* CD8^+^ T-cell stimulation from Non-Sen or Sen isografts. Experimental set-up is depicted in **a**. Cells were previously gated as CD45^+^CD3^+^CD8^+^ cells. % positive cells, as gated, are shown at the upper right corner of each plot. Representative plots are shown. (**c**) Quantification of *ex vivo* CD8^+^ T-cell stimulation flow cytometry analysis in **b**. Data are normalized based on staining in the IgG control samples, and is presented as mean % of CD8^+^IFNγ^+^ cells previously gated as CD45^+^CD3^+^ cells+s.e.m. * indicates *P* value <0.05 by Student's *t*-test. Representative experiment. *n*=4. (**d**) Schematic representation of T-cell suppression assay using CM-treated BMDM cells. (**e**) T-cell suppression assay using CM (from Non-Sen or Sen MSFs) treated naive bone marrow cells. Schematic representation of experimental set-up is shown in **d**. Control (CTR) medium consists of RPMI+5% FBS. CFSE-labelled splenocytes and unlabelled BMDM cells were plated at indicated ratios. CD8^+^ T-cell proliferation was assessed at 72 h post CD3-stimulation by flow cytometric quantification of CFSE-dilution within the CD8^+^ population of cells. * indicates *P* value <0.05 by analysis of variance. Data are presented as mean+s.d. of triplicates. Representative experiment. *n*=6.

To determine if the increases in CD11b^+^Ly6G^Hi^ and T_reg_ cells coincided with T-cell dysfunction, we assessed the ability of CD8^+^ T cells to respond to stimulation. Because there were no tumour cells and therefore no antigens present to activate the CD8^+^ T cells, we turned to a PMA (phorbol 12-myristate 13-acetate), *ex vivo*, stimulation approach. Following PMA stimulation, the cells were stained with antibodies to identify CD8^+^ T cells expressing IFNγ, a cytokine which promotes increased tumour immunogenicity and often used to assess T-cell activation^31^ (Fig. 4a, gating strategy Supplementary Fig. 4c). We found that CD8^+^ T cells harvested from senescent microenvironments exhibited reduced PMA-stimulated activation as evidenced by a significant 2.2-fold decrease in expression of IFNγ compared with non-senescent MSFs (Fig. 4b,c).

Given that senescent isografts had significantly reduced percentages of CD8^+^IFNγ^+^ cells and that CM from senescent MSFs was sufficient to alter the myeloid cells derived from naive bone marrow, we next asked if the SASP functionally impacted these cells by performing a T-cell suppression assay. Bone marrow-dervied myeloid cells (BMDMs) conditioned with senescent versus non-senescent MSF CM were plated with anti-CD3 stimulated splenocytes to assess their ability to suppress T-cell proliferation (Fig. 4d). Strikingly, myeloid cells conditioned with medium from senescent MSFs significantly suppressed CD8^+^ T-cell proliferation (Fig. 4e). Thus, SASP factors secreted by senescent stromal cells were sufficient to generate myeloid cells that potently suppress T-cell responsiveness. Given this immunosuppressive capacity, the cells formerly identified as CD11b^+^Ly6G^Hi^ will be referred to as granulocytic MDSCs (G-MDSCs)^27^,^28^. Collectively, increases in G-MDSCs and T_reg_ cells along with decreases in CD8^+^ T-cell function indicate that senescent stromal cells were sufficient to create an immunosuppressive microenvironment.

### Senescent stroma limits immune surveillance

**Figure 5:**
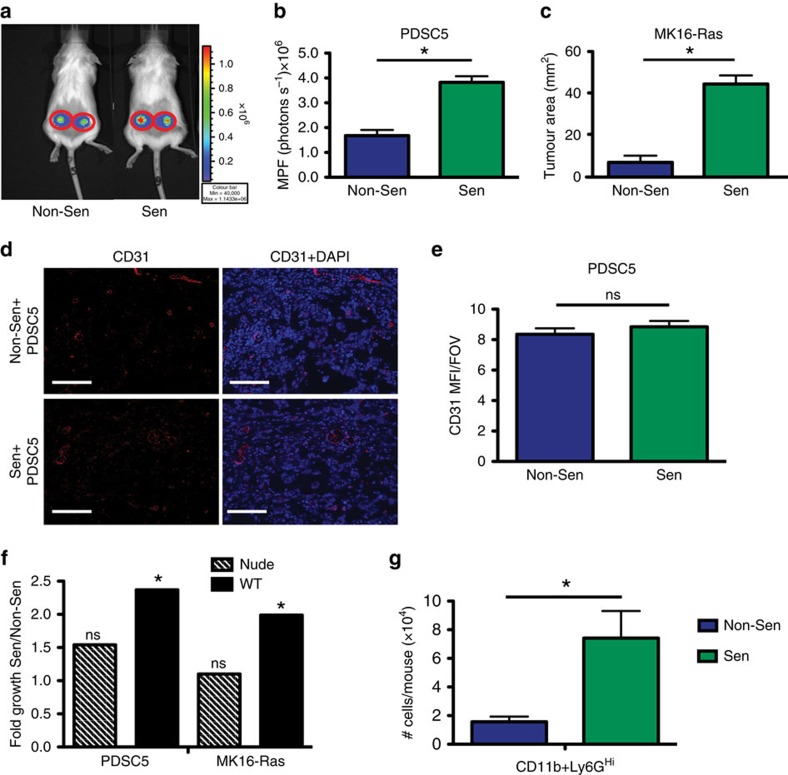
Senescent stromal cells promote immune-mediated tumour growth. (**a**) Representative images of whole-body imaging for PDSC5 tumour cell-fibroblast co-injections day 9 post injection. Scale for photon flux is shown to the right of the image. (**b**) Tumour growth following non-senescent (Non-Sen) or senescent (Sen) subcutaneous co-injections with PDSC5 tumour cells. Live imaging analysis of day 9 growth is represented as MPF (photons per second)+s.e.m. * indicates *P* value <0.05 by Student's *t*-test. *n*=8. Data shown here is derived from the same experiment shown in Fig. 6b, thus Sen mice received IgG control antibody. (**c**) Tumour growth following Non-Sen or Sen subcutaneous co-injections with MK16-Ras tumour cells. Caliper measurements were performed on day 26 following injection. * indicates *P* value <0.05 by Student's *t*-test. Data are presented as mean tumour area (mm^2^)+s.e.m. *n*=8–9. (**d**) IF CD31 (red) staining Sen versus Non-Sen subcutaneous PDSC5 tumour co-injections. Scale bar, 100 μM. Representative images. (**e**) Quantification of CD31 IF staining shown in f. ns is not significant by Student's *t*-test. Data are presented as the MFI+s.e.m. per × 20 FOV. *n*=7–8. FOV, field of view. MPF, mean photon flux. (**f**) Tumour growth following subcutaneous co-injections with PDSC5 (left) or MK16-Ras (right) tumour cells into immune-deficient, nude mice (striped bars) or immune-competent, wild-type mice (solid bars). Data represents the fold growth of Sen co-injections over non-Sen co-injections within each mouse strain. * indicates *P* value <0.05 by Wald test. ns is not significant. Data are presented as mean fold growth+s.e.m. *n*=6–10 for immune-deficient mice. *n*=6–19 for immune-competent mice. Senescent cells significantly stimulated tumour growth in wild-type mice but failed to do so in nude mice. (**g**) Flow cytometry analysis of isografts containing PDSC5 tumour cells and non-Sen or Sen-MSFs. Data represents the mean CD11b^+^Ly6G^Hi^ cell number per mouse+s.e.m., 4 days following co-injection. * indicates *P* value <0.05 by Student's *t*-test. *n*=4.

To address whether senescent stroma drives increases in tumour growth through the establishment of inflammation, we injected senescent or non-senescent MSFs admixed with one of two different isogenic tumour cell lines into wildtype mice. PDSC5 is a tumour-derived murine squamous cell carcinoma cell line and MK16-Ras is a human papilloma virus (HPV) *E6/E7* immortalized murine keratinocyte cell line that was transformed by stable transduction with *H-ras*^*v12*^. We found that senescent MSFs significantly increased the growth of each tumour cell line compared with non-senescent MSFs. Indeed, PDSC5 and MK16-Ras tumour cell growth was increased 2.3- and 6.9-fold, respectively, when either was co-injected with senescent versus non-senescent MSFs (Fig. 5a–c). This increase in growth was independent of changes in vascularization as CD31 staining revealed no significant differences between senescent and non-senescent isografts (Fig. 5d,e). Further, this was consistent with the FASST mouse model where CD31 staining was unchanged in FASST skin compared with littermate controls (Supplementary Fig. 6a–c).

To determine if this increase was due to a direct effect of senescent MSF-derived factors on tumour cells and/or input from the immune system, we co-injected tumour cells and MSFs into nude mice. While the tumours grew robustly in nude mice as expected (data not shown), we were surprised to find that the tumour promoting activities of senescent and non-senescent MSFs were equivalent (see hashed bars, PDSC5, *P*=0.24, MK16-Ras, *P*=0.49 by Wald test) (Fig. 5f). These findings deviate from previously published work^14^,^15^,^16^ and may derive from the differences in both the SASP factors expressed and the responding tumour cell types interrogated. Despite this difference, these data clearly suggest that the growth promoting effects of senescent fibroblasts in immune competent mice are derived from their ability to manipulate the immune system to favour tumour cell growth. In support of this hypothesis, we found that within tumours CD11b^+^Ly6G^Hi^ cells were increased when tumour cells were co-injected with senescent MSFs versus non-senescent MSFs (Fig. 5g).

### Senescent stroma establishes a tumour permissive environment

**Figure 6:**
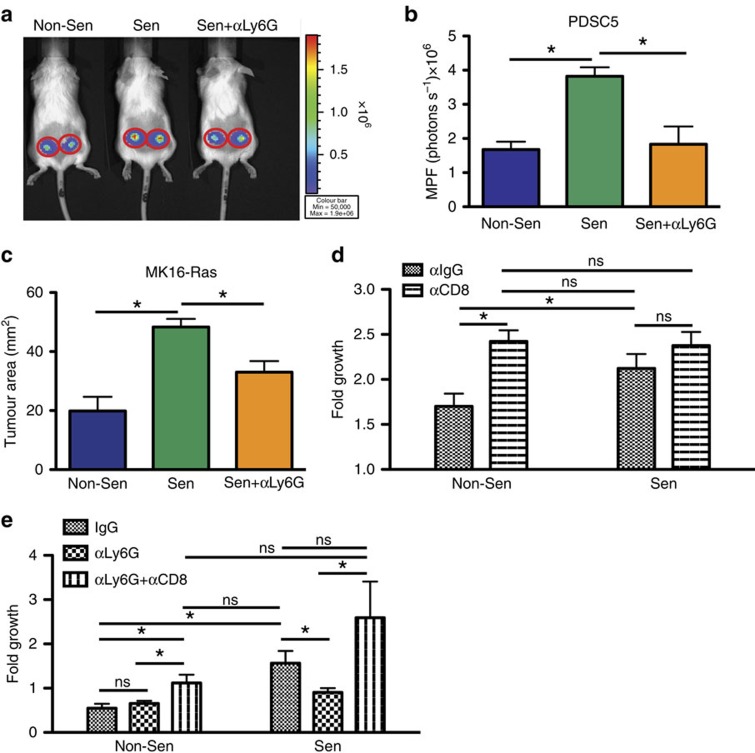
Senescent-stromal driven increases in G-MDSCs suppress CD8^+^ T cells and promote tumour growth. (**a**) Representative whole body images of PDSC5 tumour cells co-injected with MSFs as indicated following Ly6G depletion (or control). Non-senescent (Non-Sen) MSFs with PDSC5 cells (left), senescent MSFs (Sen) with PDSC5 cells (middle), and senescent MSFs with PDSC5 cells depleted of Ly6G^+^ cells (Sen+αLy6G) (right) on day 9 post injection. Sen mice received IgG antibody as a control. Scale for photon flux is shown to the right of the image. (**b**) Effect of Ly6G^+^ cell depletion on tumour growth in Non-Sen or Sen subcutaneous co-injections with PDSC5 tumour cells. Mice were treated I.P. with a neutralizing Ly6G antibody (or control IgG) every 3 days. Live imaging analysis of day 9 growth is represented as MPF+s.e.m. *P value <0.05, Student's *t*-test. *n*=7–8. (**c**) Effect of Ly6G^+^ cell depletion on tumour growth in Non-Sen or Sen subcutaneous co-injections with MK16-Ras tumour cells. Mice were treated I.P. with a neutralizing Ly6G antibody (or control IgG) every 3 days. Data are presented as mean tumour area (mm^2^)+s.e.m. at day 26 post injection. *P value <0.05, Student's *t*-test. *n*=14–18. (**d**) Effect of CD8 depletion on tumour growth in Non-Sen or Sen subcutaneous co-injections with PDSC5 tumour cells. Mice were treated I.P. with a neutralizing CD8 antibody (or control IgG) twice weekly. Tumour growth was assessed by live imaging analysis. Data are presented as mean fold growth+s.e.m. *P value<0.05, Student's *t*-test. ns is not significant. *n*=9–10. (**e**) CD8^+^ and Ly6G^+^ cell depletion in mice bearing PDSC5 tumour co-injections. Mice were treated I.P. with one out of three dosing regimens: IgG, αLy6G or αCD8+αLy6G. αCD8 was given twice weekly and αLy6G was given every 3 days for the duration of the experiment. Mice were subcutaneously injected with Non-Sen+PDSC5 or Sen+PDSC5 and tumour growth was assessed by live imaging analysis. Data are presented by mean fold growth+s.e.m. *P value<0.05, Student's *t*-test. ns is not significant. *n*=5–10. MPF, mean photon flux.

Senescent stromal-derived SASP is sufficient to generate increases in G-MDSCs that limit T-cell function (Fig. 4e). To determine if the G-MDSC population was actively limiting T-cell responses and promoting tumour growth in senescent microenvironments, we used a Ly6G neutralizing antibody (αLy6G, clone 1A8) to deplete G-MDSCs (our depletion approach efficiently eliminated greater than 85% of these cells (data not shown)) and co-injected senescent MSFs and PDSC5 or MK16-Ras cells. We found that the depletion of Ly6G^+^ cells significantly reduced the ability of senescent MSFs to promote the growth of both MK16-Ras and PDSC5 tumour cells (Fig. 6a–c). Thus, G-MDSCs are a critical mediator of senescent stromal-driven tumour promotion. In addition, using a neutralizing antibody against CD8α, we tested the consequences of a cytotoxic T lymphocyte (CTL) response on tumour growth. Upon CTL depletion, tumour growth within a non-senescent microenvironment mimicked the growth observed in a senescent microenvironment (Fig. 6d). Importantly, the senescent microenvironment showed no significant change in tumour growth following CTL depletion consistent with a microenvironment devoid of anti-tumour T-cell activity (Fig. 6d).

To determine if G-MDSCs exerted their pro-tumorigenic activity by limiting CTL responses, we performed depletion studies targeting both G-MDSCs and CTLs. Mice were treated with control antibody (IgG), Ly6G neutralizing antibody (αLy6G), or both αLy6G and αCD8. Following depletion, mice were injected with non-senescent or senescent MSFs and PDSC5 tumour cells and tumour growth was assessed. As expected, we found that in non-senescent microenvironments, depletion of Ly6G^+^ cells had no effect on tumour cell growth while co-depletion of CTLs led to significant increases in tumour cell growth when compared with control antibody (Fig. 6e). Together these data demonstrate that in the presence of non-senescent MSFs, a potent CTL response limits tumour cell growth. In contrast, in the presence of senescent MSFs where we find increases in G-MDSCs and reduced CTL activation (Figs 3b,c and 4c–e), depletion of Ly6G^+^ cells resulted in significant reductions in tumour cell growth (Fig. 6b,c,e). To establish that G-MDSCs actively limit an effective CTL response, we depleted both CD8^+^ and Ly6G^+^ cells from mice injected with senescent MSFs and tumour cells. Strikingly, we found that simultaneous depletion of CD8^+^ and Ly6G^+^ cells restored the increased tumour growth observed in the presence of senescent MSFs (Fig. 6e). These results show that senescent stromal cells mediate tumour cell growth by actively inhibiting anti-tumour CTL responses.

### SASP-derived IL-6 promotes a suppressive environment

**Figure 7:**
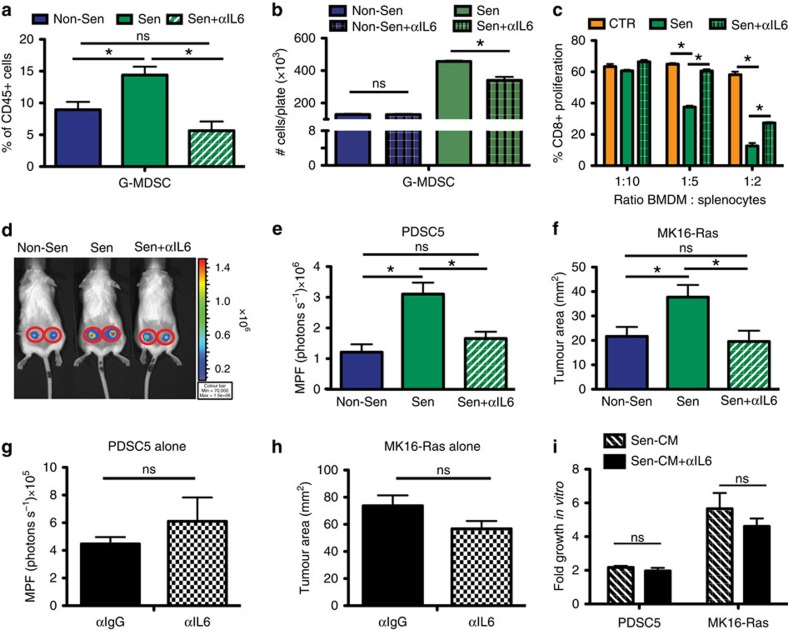
IL-6 within senescent microenvironments mediates immunosuppression and tumour promotion. (**a**) Flow cytometry for G-MDSC following IL-6 depletion in non-senescent (Non-Sen) or senescent (Sen) isografts. *P value <0.05, Student's *t*-test. ns is non-significant. Data are mean % of CD45^+^ population+s.e.m. *n*=3–7. (**b**) Flow cytotmetry of bone marrow cells treated with CM depleted of IL-6. Set-up shown in Fig. 3j. *P value <0.05, Student's *t*-test. ns is not significant. Data are mean # of cells per plate+s.e.m. *n*=4. (**c**) T-cell suppression assay following IL-6 depletion. Set-up shown in Fig. 4d. Bone marrow cells were conditioned with Sen+IgG (Sen), Sen+αIL6 or control (CTR) medium. *P value <0.05, by analysis of variance. Data are mean % proliferation+s.d. of triplicates. *n*=3. (**d**) Imaging following IL-6 depletion in PDSC5 tumour co-injections. Non-Sen with PDSC5 (Non-Sen), Sen+IgG with PDSC5 (Sen) and Sen+αIL6 with PDSC5 on day 9 post injection. (**e**) PDSC5 tumour growth in IL-6 depleted Non-Sen and Sen isografts. Data are represented as MPF (photons per second)+s.e.m. *P-value<0.05, Student's *t*-test. ns is not significant. *n*=10. (**f**) MK16-Ras tumour growth in IL-6-depleted Non-Sen and Sen isografts. *P value <0.05, Student's *t*-test. ns is not significant. Data are mean tumour area (mm^2^)+s.e.m. *n*=7–22. (**g**) Depletion of IL-6 has no direct effect on PDSC5 tumour growth when stromal cells are not present. Data are MPF+s.e.m. ns is not significant, *P*⩾0.05, Student's *t*-test. *n*=8–10. (**h**) Depletion of IL-6 has no direct effect on MK16-Ras tumour growth when stromal cells are not present. Data are mean tumour area (mm^2^)+s.e.m. ns is not significant, *P*⩾0.05, Student's *t*-test. *n*=13–15. (**i**) IL-6 neutralization does not impact *in vitro* tumour cell growth. Sen CM depleted of IL-6 or treated with IgG do not differentially impact PDSC5 or MK16-Ras growth. Data are mean fold growth+s.e.m. ns is not significant, Student's *t*-test. *n*=6. MPF, mean photon flux.

Senescent MSFs express a large cadre of inflammatory SASP factors, including IL-6 which is the most highly upregulated factor in both our MSF gene expression analysis and *in vivo* in the isograft gene expression profile (Figs 1c, 3i and Supplementary Fig. 2b). While many of the SASP factors could influence the immune response, we chose to focus on the role IL-6 might play because it has been shown to affect myeloid cell function. Indeed, IL-6 can collaborate with GM-CSF to induce suppressive myeloid cells from naive bone marrow in mice and from peripheral blood mononuclear cells in humans^32^,^33^. In addition, IF staining in FASST mouse skin shows significant increases in stromal IL-6 (Supplementary Fig. 7a,b). Thus, to determine if stromal-derived IL-6 plays a role in the inflammatory phenotypes we observed in senescent microenvironments, we depleted it *in vivo*. Senescent or non-senescent MSFs were subcutaneously injected into control or IL-6-depleted mice. In mice harbouring senescent MSFs, depletion of IL-6 reduced G-MDSCs to levels observed in mice bearing non-senescent MSFs (Fig. 7a). Consistent with the *in vivo* results, when IL-6 was neutralized in senescent CM, G-MDSCs were significantly reduced (Fig. 7b), indicating that IL-6 is important for their accumulation in the presence of senescent MSFs.

To establish that senescent-derived IL-6 was necessary to drive the emergence of MDSCs, we carried out a T-cell suppression assay using BMDMs conditioned with medium from senescent cells following IL-6 neutralization. As expected, cells grown in senescent CM neutralized of IL-6 failed to suppress T-cell proliferation to the same degree as control IgG-treated senescent CM (Fig. 7c). Together these data demonstrate an integral role for senescent stromal-derived IL-6 in the establishment of immunosuppressed microenvironments.

To test whether senescence-derived IL-6 promotes tumour cell growth through MDSCs accumulation, we co-injected tumour cells with non-senescent or senescent MSFs into mice that were treated with an IL-6 neutralizing antibody. We found that IL-6 neutralization led to a significant decrease in PDSC5 and MK16-Ras tumour cell growth relative to control IgG (1.8- and 1.9-fold, respectively) (Fig. 7d–f). Importantly, IL-6 neutralization had no effect when tumour cells were injected alone (Fig. 7g,h). Further, IL-6 neutralization of senescent CM failed to impact the *in vitro* growth of tumour cells alone (Fig. 7i). Together, these findings suggest that senescent stromal-derived IL-6 promotes tumour growth by driving accumulation of G-MDSCs.

**Figure 8:**
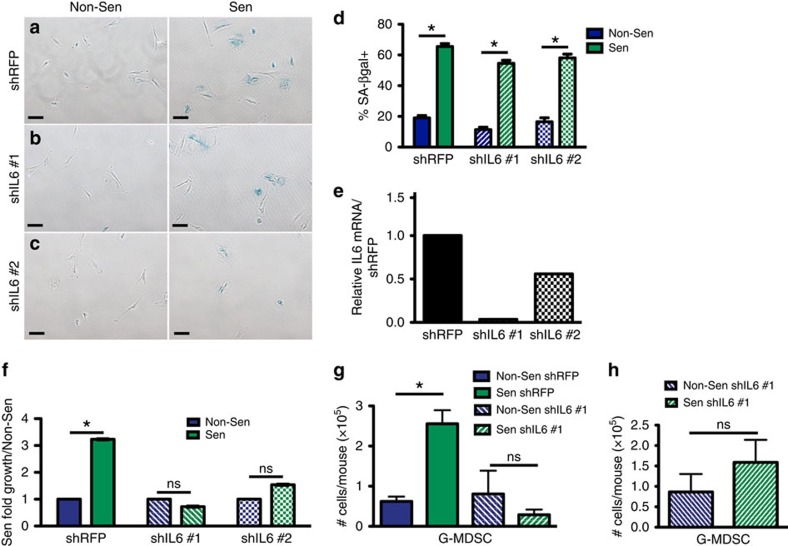
Senescence-derived IL-6 is necessary to drive G-MDSC accumulation and tumour promotion. (**a**) Senescence-associated β-galactosidase (SA-βgal) staining (blue) of shRFP-expressing MSFs treated with 10 μM TAM (Sen) or vehicle (Non-Sen) revealed ∼65% of cells were senescent following TAM treatment. Scale bar, 100 μm. *n*=3. (**b**) SA-βgal staining (blue) of shIL6#1-expressing MSFs treated with 10 μM TAM (Sen) or vehicle (Non-Sen) revealed ∼55% of shIL6 #1 cells were senescent following TAM treatment. Scale bar, 100 μm. *n*=3. (**c**) SA-βgal staining (blue) of shIL6#2-expressing MSFs treated with 10 μM TAM (Sen) or vehicle (Non-Sen) revealed ∼55% of shIL6 #2 cells were senescent following TAM treatment. Scale bar, 100 μm. *n*=3. (**d**) Quantification of SA-βgal-positive shRFP-, shIL6 #1-, and shIL6 #2-expressing cells shown in **a**–**c**. Data are mean %-positive cells+s.e.m. *P value <0.05, Student's *t*-test. *n*=3. (**e**) qRT–PCR for IL-6 mRNA expression. Data are relative IL-6 mRNA in Sen/Non-Sen shIL6 lines compared with shRFP. *n*=3. Representative experiment. (**f**) Impact of senescent stromal-derived IL-6 on tumour growth. MSFs expressing IL-6-specific hairpins (shIL6 #1 or shIL6 #2) were co-injected with PDSC5 tumour cells. shRFP was used as a control. Data are mean fold growth of Sen co-injections over Non-Sen co-injections+s.e.m. Non-Sen injections are normalized to 1. *P value <0.05, Wald test. ns is not significant. *n*=5–10. (**g**) Flow cytometry of shRFP or shIL6 #1-expressing Non-Sen or Sen MSF isografts. Mice were injected with senescent or non-senescent MSFs that expressed a control shRFP or were depleted of IL-6 through the expression of the shIL6 #1 short hairpin. Data represents the mean number of G-MDSCs+s.e.m., per mouse. *P value <0.05, Student's *t*-test. ns is not significant. *n*=3–4. (**h**) Flow cytometry of shIL6 expressing Non-Sen or Sen MSF-PDSC5 co-injections. Mice were injected with PDSC5 tumour cells and senescent or non-senescent MSFs depleted of IL-6 through the expression of shIL6 #1. Data represents the mean number of G-MDSCs+s.e.m., per mouse. ns is not significant, Student's *t*-test. *n*=3–4. qRT–PCR, quantitative PCR with reverse transcription.

To establish that the source of IL-6 was indeed senescent stromal cells and not another cell type within the microenvironment, we engineered two different IL-6-targeting short hairpin MSF cell lines. When control shRFP-expressing MSFs were induced to senesce, they stained positive for SA-βgal, expressed increased levels of IL-6, (Fig. 8a–e) and upon co-injection with PDSC5 tumour cells, promoted tumour cell growth 3.2-fold compared with non-senescent, shRFP-expressing MSFs (Fig. 8f). In contrast, the senescent shIL-6 MSF lines, which expressed significantly reduced levels of IL-6 (Fig. 8e), failed to promote tumour cell growth compared with non-senescent MSFs expressing the same hairpins (Fig. 8f). This lack of growth promotion corresponded to the degree of IL-6 knockdown in the individual cell lines with shIL6 #1 showing the greatest degree of IL-6 knockdown (Fig. 8e) and the greatest reduction of tumour growth promotion compared with the control shRFP line (Fig. 8f). Importantly, when the inflammatory infiltrate was profiled, the presence of control senescent shRFP-expressing MSFs led to increases in G-MDSCs while shIL6-expressing senescent MSFs failed to enrich for this population (Fig. 8g) even in the presence of tumour cells (Fig. 8h). Together these data indicate that senescent stromal-derived IL-6 plays a key role in the establishment of an environment that potently inhibits T-cell function through G-MDSCs.

### Senescence-associated inflammation increases in human skin

**Figure 9:**
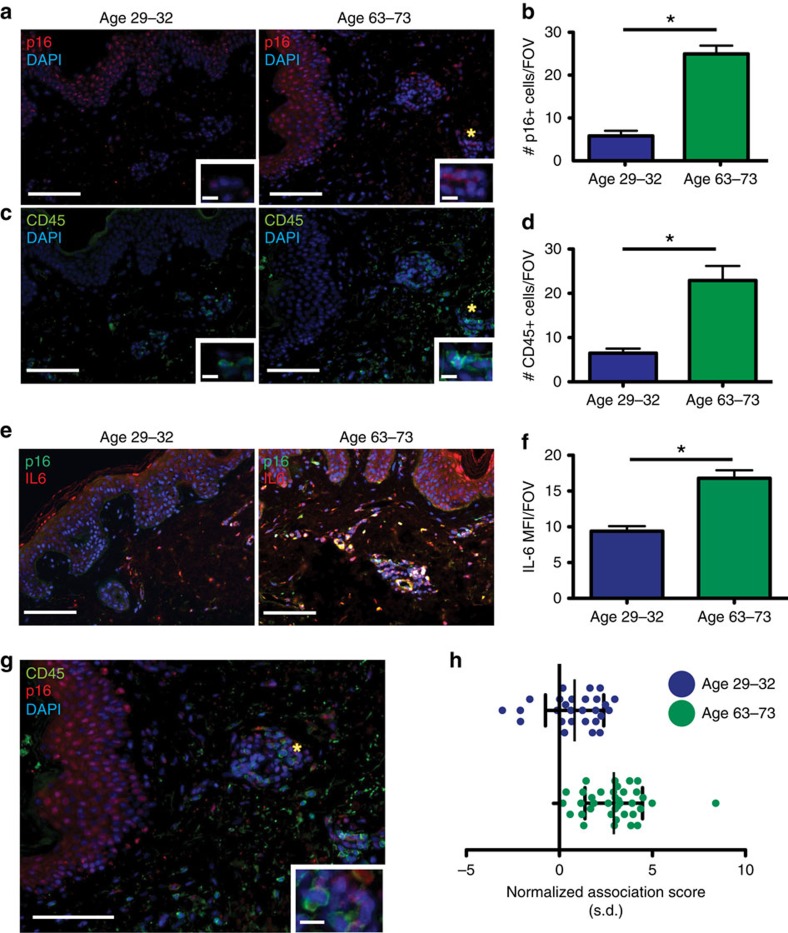
Increases in senescent stromal cells in aged human skin correlate with increases in inflammation. (**a**) IF staining for p16^INK4a^ (red) in human skin samples grouped by age. Nuclei are DAPI-stained (blue). Scale bar, 100 or 10 μm in zoomed inset image. *site of zoomed image. (**b**) Quantification of p16^INK4a^ (red) staining shown in a. grouped by age. *P value <0.05, Student's *t*-test. Data are mean+s.e.m. Graph contains data from four donors per age group, multiple images (5–17) per donor. (**c**) IF staining for CD45 (green) in human skin samples grouped by age. Nuclei are DAPI-stained (blue). Scale bar, 100 or 10 μm in zoomed inset image. *site of zoomed image. (**d**) Quantification of CD45 (green) staining shown in **c**. grouped by age. *P value <0.05, Student's *t*-test. Data are mean+s.e.m. Graph contains data from four donors per age group, multiple images (5–17) per patient. (**e**) IF staining for p16^INK4A^ (p16, green) and IL-6 (red) on human skin. Nuclei are DAPI-stained (blue). Secondary alone control showed no detectable staining (data not shown). Double-positive staining (yellow) was noted in older individuals (Age 63–73). Scale bar, 100 μm. (**f**) Quantification of IL-6 IF staining shown in **e**. Data are the MFI+s.e.m. per × 20 FOV. *P value <0.05, Student's *t*-test. Four donors per age group, multiple images (4–10) per donor were analysed. (**g**) IF co-staining for p16^INK4a^ (red) and CD45 (green) in human skin. Nuclei are DAPI-stained (blue). Panel **e** is the merged image of the staining shown in **a**,**c**. Scale bar, 100 or 10 μm in zoomed inset image. *site of zoomed image. (**h**) Images from staining shown in **e** were analysed using the same spatial statistics described in Fig. 1g. In samples from donors aged 63–73, a strong association of p16^+^ and CD45^+^ cells was observed (mean normalization score 2.9, 95% CI 2.4–3.4). Samples from donors aged 29–32 show weak association (mean normalization score 0.8, 95% CI 0.2–1.4). 4 patients per age group. Error bars are s.d. *n*=27–37. FOV, field of view.

Senescent cells increase in many tissues including the skin of humans as they age^7^. In addition, recent evidence shows that suppressive myeloid cells increase with age in the circulation of humans and are elevated within the spleens of older mice^6^,^34^,^35^. However, whether the accumulation of suppressive myeloid cells within senescent tissues of older individuals can precede the appearance of a tumour remains an open question. To identify senescent cells within human skin, we turned to the well-characterized senescence maker p16^INK4a^ (p16), which is used extensively in human tissue^23^,^36^. We co-stained normal human skin of varying age with antibodies against p16 (red) and CD45 (green) to identify senescent cells and leukocytes, respectively. Our staining approach revealed a pattern that was consistent with that observed in the FASST mouse model. Intriguingly, we found that the skin from older individuals aged 63–73 years had a 4.3-fold increase in senescent cells within the stroma (Fig. 9a,b) that corresponded with a 3.5-fold increase in CD45^+^ cells (green) (Fig. 9c,d) compared with skin from younger individuals aged 29–32 years. Additionally, using IL-6 (red)/p16 (green) co-staining we provide evidence that senescent cells within human tissue express the important inflammatory mediator IL-6, which can also serves as a surrogate for SASP expression. We detected p16^+^IL-6^+^ cells in the older (age 63–73) cohort of donors as well as significant increases in IL-6 within the stromal compartment (Fig. 9e,f). These finding demonstrate that within aging human skin, senescent stromal cells express IL-6 and may affect local inflammation.

We next determined whether the presence of senescent stromal cells correlated with changes in immune localization within human skin. Thus, we returned to p16 and CD45 co-staining and performed the same non-biased spatial analysis previously used in the FASST mouse skin (Fig. 1f,g). Strikingly, our spatial analysis from individuals aged 63–73 years revealed a clear preferential localization of CD45^+^ cells near p16^+^ senescent stromal cells (values>0) reminiscent of that observed in the skin of FASST mice (Fig. 9g,h). This strong preferential localization was not observed in the younger individuals (Fig. 9h). This finding suggested that within aging human skin, senescent stromal cells reshape the local immune landscape.

**Figure 10:**
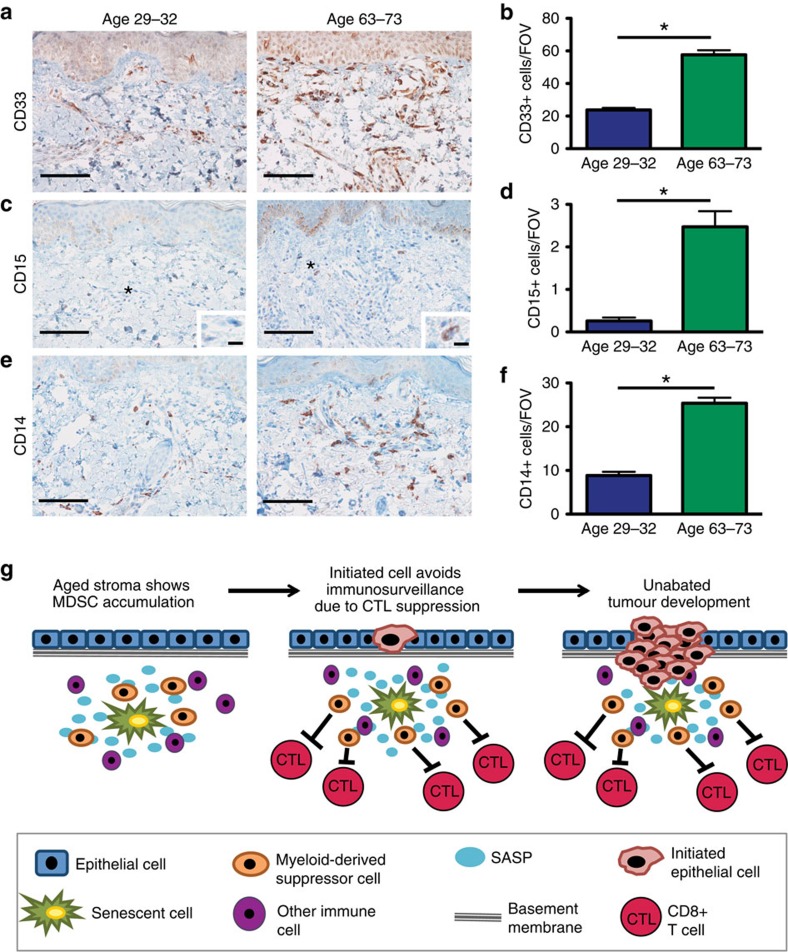
Immunosuppressive cell types are enriched in aged human skin. (**a**) Representative IHC staining for CD33 in human skin grouped by age. Scale bar, 100 μm. (**b**) Quantification of CD33 IHC staining in human skin grouped by age. * indicates *P* value <0.05 by Wald test. Data are presented as mean CD33^+^ cells per × 20 FOV+s.e.m. Data represents four donors per age group, multiple images (5–27) per donor were used for quantification. (**c**) Representative human IHC staining for CD15 grouped by age. Scale bar, 100 or 10 μm for zoomed inset image. * indicates location of zoomed image. (**d**) Quantification of CD15 IHC staining in human skin grouped by age. * indicates *P* value <0.05 by Wald test. Data are presented as mean CD15^+^ cells per × 20 FOV+s.e.m. Graph is of four donors per age group, multiple images (2–15) per donor were used for quantification. (**e**) Representative human IHC staining for CD14 grouped by age. Scale bar, 100 μm. (**f**) Quantification of CD14 IHC staining in human skin grouped by age. * indicates *P* value<0.05 by Wald test. Data are presented as mean CD14^+^ cells per × 20 FOV+s.e.m. Graph is of four donors per age group, multiple images (5–22) per donor were used for quantification. (**g**) Model for SASP-driven immunosuppression resulting in age-related tumour development. Senescent stromal cells accumulate in a mosaic fashion in aged stromal compartments in the absence of tumours. Through the SASP, senescent fibroblasts are sufficient to drive increases in inflammation that is characterized by enrichment of MDSCs. These MDSCs suppress anti-tumour CD8^+^ T-cell (CTL) responses and promote incipient tumour outgrowth. FOV, field of view.

We next sought to determine if the CD45^+^ cells observed within the skin of older individuals expressed cell surface markers consistent with immunosuppressive cell types. CD33 marks human cells analogous to MDSCs found in mice^37^. IHC staining for CD33 revealed a marked increase in CD33^+^ cells in the older age grouping compared with the younger (Fig. 10a,b). The cell surface proteins CD15 and CD14 can be used to further identify granulocytic and monocytic cells, respectively^37^. Further staining indicated that cells positive for CD15 were also increased in the skin of older individuals, as were CD14^+^ cells (Fig. 10c–f). Importantly, these normal skin samples had no detectable hyperplasia or neoplasia that could explain the increased presence of CD33^+^ MDSCs. Thus, these data taken together suggest that senescent cells within older individuals may drive the development of inflammatory microenvironments that shelter incipient tumour cells from effective CTL detection and elimination (Fig. 10g).

## Discussion

Senescence plays an important role in biological processes ranging from patterning in developing embryos and wound healing, to tumour prevention and tumour promotion^38^,^39^,^40^,^41^,^42^,^43^,^44^. The mechanisms responsible for the paradoxical role that senescence plays in tumorigenesis have remained obscure but are likely to lie in the senescent secretory profile that is defined by the cell of origin^11^. While a recently published report has implicated transformed senescent cells in the establishment of immunosuppression^30^, our work is the first to identify senescence as a driver of immunosuppression distinct from the role played by tumorigenic cells. In our work, we found that the stromal-derived SASP-factor IL-6 establishes myeloid-driven immunosuppression where CD8^+^ T cells were inhibited, resulting in unrestrained tumour growth. Importantly, senescent stromal cells were sufficient to drive these changes. This phenotype is in contrast to the anti-tumorigenic role senescence plays when it arises in tumour cells^13^,^45^. In addition to directly limiting replicative capacity, in this setting the secretion of inflammatory cytokines can elicit immune-mediated clearance of senescent tumour cells^46^. Indeed, the immune-mediated clearance of senescent tumour cells has been documented in several settings. Xue *et al*. demonstrated that within the liver, senescent carcinoma cells elicited immune-mediated clearance that restrained tumour growth^13^. In contrast, in an obese setting, senescent liver stellate cells drove promotion of liver carcinomas^47^. Conversely, senescence within premalignant hepatocytes induced CD4^+^ T-cell-mediated clearance and thus limited liver cancer development^45^. In a PTEN-null, prostate cancer model, again robust induction of senescence was observed within tumour cells throughout the tissue^30^. However, these cells were not cleared, suggesting that clearance of senescent cells may be tissue-specific, cell type-specific and/or specific to the mutational profile of the transformed cells in question.

Despite the tumour suppressive role senescence plays when it arises within incipient tumour cells, a recent study demonstrated that senescence represents an important tumour-promoting mechanism in aging animals. In this study, it was shown that the elimination of senescent cells within an aging mouse decreased life long tumour rates^10^. Our work provides a possible mechanism to describe these findings. Indeed we demonstrate that senescent stromal cells create an immunosuppressive environment characterized by the accumulation of MDSCs that limit CD8^+^ T-cell responses and allow tumour cell growth.

To date, investigation into the mechanisms that drive MDSC recruitment to a premalignant or malignant tissue has been restricted to determining how tumour cells (or premalignant cells) achieve this. Our model is distinct because it demonstrates that age-related changes in the stromal compartment that arise in the absence of premalignant and malignant cells are sufficient to drive the recruitment of suppressive cells. Because the presence of senescent stromal cells correlates with increases in MDSCs in aged human skin, our findings suggest that senescent stromal cells provide a safe harbour for emerging neoplastic clones. Indeed, it is possible that the genesis of many tumours lies in the creation of a perfect storm where initiated tumour cell clones arise within the immunosuppressive sphere of senescent stromal cells. As such, our findings raise the possibility that senescent stromal cells represent an important preventative target.

While our model is representative of the stromal compartment of aged tissue (Fig. 9) and shows that senescent stromal cells can recruit suppressive cells that support tumour formation, it raises the question, do all senescence inducers within the stromal compartment induce similar changes? In fact it is unlikely that all inducers of senescence invoke identical immune changes. For example, Campisi and colleagues have shown that unlike chronologically, ARF, or Ras-induced senescence, p16-induced senescence does not lead to SASP expression^48^. Thus, one might expect that p16-induced senescence would not impact the local immune response as we have described for p27-induced senescence, which induces a SASP reminiscent of that observed in fibroblasts undergoing chronologic aging^11^. Finally, it will be important that future studies test the impact of senescent stromal cells in a spontaneous tumour model. While our isograft model was an important tool to understand how senescent fibroblasts can induce local immune changes, the real challenge will be to incorporate these findings into a spontaneous model where senescent fibroblasts arise in the direct vicinity of initiated, preneoplastic cells. The challenge now lies in the design of an inducible model that maintains the mosaic activation of senescence in the stromal compartment reminiscent of what we (Fig. 9) and others^7^ have reported in human tissue and the simultaneous induction of initiating mutations in the direct vicinity of senescent stromal cells and their associated immune changes. Such models await the development of stromal specific inducible alleles that can be combined with inducible tumour initiating epithelial alleles. Such models will be critical for the future development of multi-pronged cancer therapy.

## Methods

### Mice

Wildtype FVB, female mice age 6–8 weeks were used in all subcutaneous injection and co-injection experiments unless otherwise noted. For naturally aged mouse experiments, 24-month-old and 2-month-old, wild-type, female Balb/c mice were obtained from the National Institute of Aging. All mice were used in compliance with the Washington University Institutional Animal Care and Use Committee under protocol #20130196.

To obtained stromal-specific activation of a lox-stop-lox cassette mice expressing a chimaeric gene encoding the Cre-ER^T2^ fusion protein, which allows for TAM-dependent recombination under the control of a mesenchymal-specific regulatory sequence derived from the pro-alpha 2(I)collagen gene (Col-Cre-ER^T2^ mice, kind gift of Benoit de Crombrugghe (M.D. Anderson Cancer Center) and Christopher Denton (Royal Free and University College Medical School) were crossed with mice that conditionally express the cell cycle inhibitor, *p27*^*Kip1*^ from the ROSA26 locus (ROSAlox-stop-lox-*p27*^*Kip1*^) (ref. 22). Finally, mice were subject to speed congenics and bred to an FVB background. For transgene activation, four-week-old FASST female mice and control littermates were given TAM (5 mg per 10 g body weight) by intraperitoneal (IP) injection twice.

Mouse genotyping was carried out as follows: genomic DNA was extracted from mouse tissue using the RED Extract N Amp kit (XNAT, Sigma). Primer sequences used to determine the presence of the *p27*^*Kip1*^ transgene within the ROSA26 locus were as follows: the wildtype ROSA26 locus was amplified using 3′ wt: 5′- GGAGCGGGAGAAATGGATATGAAGTACTGG -3′ (forward) and 5′: 5′- CAAAGTCGCTCTGAGTTGTTATCAGTAAGG -3′ (reverse) and the *p27*^*Kip1*^ containing locus was amplified using 3′ wt: 5′- GGAGCGGGAGAAATGGATATGAAGTACTGG -3′ (forward) and 3′delta: 5′- TCCAAGAGTACTGGAAAGACCGCGAAGAGT -3′ (reverse). Primer sequences used to amplify the Cre-ER^T2^ transgene were 3′: 5′- ATCCAGGTTACGGATATAGT -3′ (forward) and 5′: 5′- ATCCGAAAAGAAAACGTTGA -3′ (reverse).

To amplify DNA of interest, 1 μl DNA extract was combined with 9 μl of master mix (XNAT kit with 0.5 μM appropriate forward and reverse primers) in PCR tubes and amplified for one cycle at 94 °C for 1 min followed by 35 cycles at (94 °C for 30 s, 58 °C for 60 s and 72 °C for 60 s) and a 4 °C hold. PCR products were resolved on a 1% agarose/TAE (Fisher Scientific) containing 0.002% SyberSafe (UV gel stain, Invitrogen) gel at 100 volts for 45 min, and visualized with UV light.

### Cell lines and conditioned media treatments

The MSF cell line was isolated by removing the dorsal skin from a female FASST mouse. The tissue was minced, then transferred to a conical tube containing collagenase solution (2 mg ml^−1^ Collagenase A, 1 mg ml^−1^ hyaluronidase, 2 U ml^−1^ DNase I in serum-free DMEM). The skin was digested at 37 °C on a rotisserie for 4 h. Following digestion, the cells were plated and 24 h later the non-adherent cells were removed.

The PDSC5 cell line was a kind gift from Dr Lisa Coussens (Oregon Health Science University) and was stably transduced with click-beetle red (CBR) luciferase. MK16 cells are a primary mouse keratinocyte cell line immortalized with HPV *E6/E7* and were a kind gift from Dr Craig Woodworth, Clarkson University. The MK16 cells were stably transduced with *H-Ras*^*v12*^ and CBR to generate the MK16-Ras cell line. All cell lines were regularly tested for mycoplasma but were not authenticated.

To induce senescence *in vitro*, MSFs were treated with 10 μM TAM for 48 h. Control MSFs were treated with ethanol as vehicle. Upon treatment, cells were moved to a modular incubation chamber and cultured at 37 °C in 3% oxygen, 5% carbon dioxide, balanced with nitrogen.

Conditioned media (CM) was generated using MSFs isolated from FASST mice as described. The MSFs were induced to senesce as described. At day 9, CM was generated by washing the plates and placing serum-free medium (RPMI 1640+1% penicillin/streptomycin) onto confluent plates of control or senescent MSFs. The medium was collected after 24 h.

IL-6 neutralization of CM was performed by the use of an IL-6 neutralizing antibody. Following collection of the CM from senescent or control MSF cells, IL-6 neutralizing antibody or IgG control antibody was added to the CM at a concentration of 5 μg ml^−1^.

### Plasmids and virus production

The RNAi constructs were generated by cloning IL-6 hairpins into a pLKO.1-hygromycin plasmid using the Age1 and EcoR1 sites. The following are sequences for the RNAi constructs targeting IL-6 or a non-targeting control (shRFP): pLKO.1-shRFP-hygro 5′- CTCAGTTCCAGTACGGCTCCA -3′, pLKO.1-shIL6 #1-hygro 5′- GCAATGGCAATTCTGATTGTA -3′, pLKO.1-shIL6 #2-hygro 5′- CCAATGCTCTCCTAACAGATA -3′. MSCV-Cre-ER^T2^-puro was obtained from Dr Emily Cheng (Memorial Sloan-Kettering). PDSC5 and MK16-Ras tumour lines were transduced with pBabe-Click-Beetle Red (CBR)-hygro and pBabe-H-ras^V12^-puro was stably transduced into MK16 keratinocytes to fully transform the cells and create the MK16-Ras line. The lentivirus production and infections were carried as described^49^.

### SA-β-gal staining

SA-βgal staining in the skin of mice was performed on frozen sections that were fixed in 0.2% glutaraldehyde for 10 min. at 4 °C. Slides were washed in PBS and then submerged in X-gal solution (1 mg ml^−1^ 5-Bromo-4-chloro-3-indolyl β-D-galactopyranoside, 150 mM NaCl, 2 mM MgCl_2_, 5 mM K_3_Fe(CN)_6_, 5 mM K_4_Fe(CN)_6_, 40 mM NaPi pH 6.0, in H_2_0). The X-gal solution was passed through a 0.45 μm filter before use to remove particulate. Slides were kept at 37 °C in the dark until the stain developed (∼6 h). After staining, the slides were fixed for 10 min in 0.2% glutaraldehyde at room temperature, washed in PBS and then the nuclei were counterstained using Nuclear Fast Red (Sigma)^49^.

### Quantitative PCR

RNA was isolated using an RNeasy isolation kit (Qiagen) and cDNA synthesis was performed using SuperScriptII reverse transcriptase (Invitrogen). Quantitative PCR was performed using Taqman probe/primer sets to amplify the following genes: GAPDH (Mm99999915_g1), IL-6 (Mm00446190_m1), CXCL5 (Mm00436451_g1), CXCL1 (Mm04207460_m1), CCL2 (Mm00441242_m1), CCL5 (Mm01302427_m1), CCL8 (Mm01297183_m1), CCL7 (Mm00443113_m1) and CXCL14 (Mm00444699_m1) (Thermo Fisher). For experiments using isograft tissue for gene expression analysis, isografts were isolated at 11 days post injection. The tissue was snap-frozen and then pulverized using a mortar and pestle. RNA isolation and cDNA synthesis was performed as above. The samples were then pre-amplified using Taqman PreAmp Master Mix kit and Taqman primers for the following genes of interest: GAPDH (Mm99999915_g1), STAT3 (Mm01219775_m1), Nos2 (Mm00440502_m1), Arg1 (Mm00475988_m1), CXCL1 (Mm04207460_m1), M-CSF (Mm00432686_m1), GM-CSF (Mm01290062_m1), IL-6 (Mm00446190_m1), CCL2 (Mm00441242_m1), TGF*β* (Mm01178820_m1), IL-1*β* (Mm00434228_m1), IL-1*α* (Mm00439620_m1) and IL-10 (Mm01288386_m1) (Thermo Fisher). All primer sequences are proprietary. For analysis, values are normalized to GAPDH.

### RNA-sequence (RNA-seq) analysis

RNA-seq reads were mapped to the mouse genome (mm9) using TopHat^50^. Mapped reads were assigned to RefSeq annotated genes using htseq-count (http://www-huber.embl.de/users/anders/HTSeq/doc/count.html). Differential expression was assessed using edgeR with TMM normalization^51^. Log2 fold changes were computed after applying a floor of 1 c.p.m. to ensure that genes with very low over all expression, but large relative fold changes did not affect the analysis.

Gene set enrichment analysis was performed as in using the log2 fold-change to rank genes. *P*-values are those reported from GSEA for the enrichment of factors in the JAK-STAT pathway.

### Enzyme-linked immunoSorbent assay (ELISA)

The protein level of IL-6 was detected in CM from non-senescent and senescent MSFs using a high-sensitivity ELISA kit (eBioscience) according to manufacturer's instructions (cat. # BMS603HS). CM was generated as stated above.

### Growth curve

Vehicle- or TAM-treated MSFs were plated on day 8 following TAM removal. Cells were plated at a concentration of 20,000 cells/well in six-well plates with each day plated in triplicate. Cells were lifted with trypsin and counted using a hemocytometer daily for the four days immediately following plating. A two-factor analysis of variance with Bonferroni's multiple comparisons post test was used to calculate statistical significance among groups. Data shown are representative of two independent experiments and presented as mean ± s.e.m.

### Immunohistochemistry and immunofluorescence staining

FASST mouse skin was stained as follows: CD45, IHC staining, was carried out on formalin-fixed, paraffin embedded skin sections. Slides were deparaffinized by heating for 20 min at 55 °C followed by submergence in a xylene bath twice for 5 min. The tissue was then rehydrated by sequential dips into baths of decreasing ethanol concentration going from 100% ethanol to 100% H_2_0. Endogenous peroxidase activity was blocked using 3% hydrogen peroxide in methanol. Following PBS wash, sections were blocked in blocking buffer (5% normal goat serum, 2.5% BSA in PBS) in a humidified chamber for 30 min at room temperature. Primary antibody (CD45, 1:500) was added following the blocking step. After 30 min, slides were washed in PBS and re-blocked for 10 min in blocking buffer. A biotinylated secondary antibody was used at 1:250 for 30 min. The slides were washed in PBS and then ABC-Elite (Vector Labs) solution was applied for 30 min. Following PBS wash, the stain was developed using DAB (Dako) and briefly washed in water before haematoxylin counterstain and mounting.

For CD45, GFP immunofluorescent co-staining, frozen skin samples were used. Skin samples were first permeabilized using 0.5% Triton-X in PBS for 10 min at room temperature. Following PBS wash, samples were blocked using Serum-free Protein Block Solution (Dako) for 30 min. Primary antibodies, anti-CD45-PE at 1:200 or anti-IgG-PE control, and anti-GFP at 1:1,000, were then added to the skin sections and the slides were placed in a humidified container at 4 °C overnight. The next day, the slides were washed in PBS and secondary antibody for detection of the unconjugated GFP antibody was added for 1 h at room temperature. The slides were washed and subsequently mounted using ProLong Gold with DAPI anti-fade mounting reagent (Life Technologies). Additional information about antibodies including their catalog numbers can be found in Supplementary Table 1.

CD31, IL-6 and Ly6G IF staining was performed on frozen FASST mouse skin and littermate controls or 2-month and 24-month-old mice where noted. The procedure was similar to what is detailed above for frozen section staining. However, a primary antibody to CD31 (1:50), IL-6 (1:50) or Ly6G (1:100) were used along with a corresponding secondary. Additional information about antibodies can be found in Supplementary Table 1. Quantification of staining was performed using Fiji software to analyse mean fluorescence intensity (MFI) per image for CD31. Before calculating the MFI, the freehand selection tool was used to select only the stromal region for MFI quantification thus excluding the epithelium from the analysis. Quantification of Ly6G staining was performed using a similar stromal selection technique.

Isograft staining was carried out as follows: formalin-fixed, paraffin embedded isografts were sectioned and stained. CD11b-PE (1:200) and Gr-1-PE (1:400) or as control IgG-PE (1:200 or 1:400), immunofluorescent staining was performed using the same procedure. Slides were warmed at 55 °C for 20 min, xylene deparaffinized and rehydrated as noted above for CD45 skin staining. Antigen retrieval using citrate was performed by boiling the slides for 10 min followed by 20 min of cooling in the citrate buffer solution. Slides were washed in H_2_O and then transferred to PBS. The slides were then blocked in a block/perm solution (3% normal goat serum, 1% BSA, 0.1% Triton-X, 0.05% Tween-20, with F_c_ blocking antibody (BD Pharmingen)) at 1:200 in PBS for 2 h at room temperature in the dark in a humidified chamber. Slides were washed in PBS and then mounted using ProLong Gold with DAPI anti-fade mounting reagent (Life Technologies).

GFP and IL-6 immunofluorescent co-staining on frozen isograft tissues was performed in a similar fashion to the GFP and CD45 co-staining detailed above in the FASST skin. Briefly, section were permeabilized using 0.5% Triton-X in PBS for 10 min at room temperature and blocked using Serum-free Protein Block Solution (Dako) for 30 min. Primary antibodies, anti-GFP at 1:1,000 and anti-IL-6 at 1:50, were then added to the skin sections for overnight incubation at 4 °C. Slides had secondary antibody applied the next day for 1 h at room temperature. The slides were mounted using ProLong Gold with DAPI anti-fade mounting reagent (Life Technologies).

CD31 immunofluorescent staining was performed on frozen isograft tissue. The procedure was similar to what is detailed above for FASST skin frozen section staining. Quantification of staining was performed using Fiji software to analyse MFI per image. Additional information about antibodies can be found in Supplementary Table 1.

For haematoxylin and eosin staining, slides were deparafinized in three changes of xylene then cleared in two changes of 100% EtOH. The slides were then dipped 10 times into 95% ethanol and placed in running tap water for 5 min. The slides were put into haematoxylin for 1 min and then under running tap water until the water ran clear. They were then placed into 95% ethanol and subsequently stained in eosin for 3 min. The slides were dipped five times in two changes of 95% ethanol and then 10 times in three changes of 100% ethanol. The slides were finally dipped 10 times in three changes of xylene and mounted.

For staining of cultured fibroblasts, MSFs were stained for FAP or phosphorylated STAT3 (p-STAT3) following senescence induction. Cells were plated on coverslips, fixed in 4% paraformaldehyde for 10 min at room temperature, washed with 1 × PBS and subsequently permeabilized using 0.5% Triton-X in PBS for 10 min at room temperature. The cells were then stained using an anti-FAP primary antibody (1:200) or anti-p-STAT3 primary antibody (1:50) followed by an appropriate secondary antibody. Additional information about antibodies can be found in Supplementary Table 1.

All human samples were obtained from a retrospective study using de-identified, formalin-fixed and paraffin-embedded human tissue specimens that were retrieved from archived tissue bank of the Washington University Dermatopathology Center in accordance with guidelines set by the Institutional Review Board of Washington University (IRB ID#: 201309067). Under this IRB protocol no informed consent was required from the patients. Human IF staining was carried out as follows: IF staining for p16^INK4a^ (p16) and CD45 were used in a co-staining procedure similar to formalin-fixed, paraffin embedded, mouse sections. Briefly, following deparaffinization and rehydration, antigen retrieval was performed by boiling slides on a hot plate and then allowing slides to cool before staining. Tissue sections were blocked and then primary antibody applied for detection of p16 and CD45. After overnight incubation with the primary antibody at 4 °C, the corresponding secondary antibodies were applied for 1 h at room temperature. Slides were then washed and mounted using ProLong Gold with DAPI (Life Technologies) Additional information about antibodies can be found in Supplementary Table 1.

Human IL-6, p16 IF co-staining used the same techniques stated for p16, CD45 staining. Quantification of staining was performed using Fiji software to analyse MFI per image. Before calculating the MFI, the freehand selection tool was used to select only the stromal region for MFI quantification thus excluding the epithelium from the analysis. Details regarding antibodies can be found in Supplementary Table 1.

Human IHC staining was carried out using the following antibodies: CD33 (clone: PWS44, Leica Biosystems, cat. # NCL-L-CD33), CD15 (clone: MY-1, Abcam, cat. # ab754), CD14 Ab-2 (clone: 7, Thermo Fisher Scientific, cat. #. MS-1080), p27 (clone: G173–524, BD Pharmingen, cat. # 554069). Slides were heated to 60–70 °C for 30 min and then deparaffinized in xylene three times, 5 min each. Tissue was rehydrated through incubation in decreasing grades of ethanol up to H_2_O. Antigen retrieval was achieved through incubating slides in citrate buffer in a pressure cooker (125 °C for 1 min then 95 °C for 20 min, CD33, CD15, CD14), or boiled for 10 min on a hot plate (p27). Slides were cooled for 20 min and then peroxidase activity was blocked using 3% H_2_O_2_ in PBS for 10 min at room temperature. Slides were rinsed in PBS and blocked using Dako Serum-free Protein Block (Dako) for 30 min at room temperature in a humidified chamber. Following blocking step, primary antibody was diluted in Dako Antibody Diluent (Dako) and applied to the slides at an appropriate concentration (CD33 1:100, 4 μg ml^−1^, CD15 1:10, not purified, CD14 1:20, not purified, p27 1:50, 10 μg ml^−1^). Slides were incubated on primary antibody overnight at 4 °C in a humidified chamber. The next day, slides were washed in PBS and a biotinylated, goat anti-mouse secondary (Vector Labs, Cat. #. BA-4001, 6 μg ml^−1^) diluted in Dako Antibody Diluent was placed on the slides for 30 min at room temperature. Slides were washed in PBS and ABC-Elite solution (Vector Labs) was then applied for 30 min at room temperature. Slides were washed in PBS and DAB (Dako) was used to develop the stain. Slides were counterstained using haematoxylin for 5 min, dipped in bluing reagent 10 times before being rinsed for 2 min in running water. Tissue sections were dehydrated by increasing grades of ethanol, cleared with xylene and then mounted. Additional information about antibodies can be found in Supplementary Table 1.

### Microscopy

All bright-field images were acquired using a conventional Nikon microscope and Nikon Elements imaging software. All fluorescent images were acquired by a Nikon Eclipse Ti-E microscope with automated stage and focus control driven by Metamorph Software.

### Bone marrow isolation and conditioned media treatment

Bone marrow was isolated from the long bones of wildtype FVB mice via centrifugation. The bone marrow cells were then cultured in RPMI media+5% heat-inactivated FBS (Sigma) on petri dishes in a 37 °C tissue culture incubator at 5% oxygen, 5% carbon dioxide, balanced with nitrogen. To assess the effects of conditioned media, the bone marrow cells were plated at 2.5 × 10^5^ cells per 10 cm dish in either control RPMI+5% heat-inactivated FBS (Sigma) medium, senescent MSF CM+5% heat-inactivated FBS (Sigma), or control MSF CM+5% heat-inactivated FBS (Sigma). For assessing the contribution of IL-6 in this assay, bone marrow cells were plated with either control media (RPMI media+5% heat-inactivated FBS) or media supplemented with IL-6 (40 ng ml^−1^) and GM-CSF (40 ng ml^−1^). IL-6 neutralized versions of the CM was made as described and used where noted. Cells were cultured in the above conditions for 4 days before flow cytometry analysis.

### *In vitro* T-cell suppression assay

The suppression assay was modified from published^52^ work by first isolating bone marrow cells and treating them with senescent or non-senescent MSF CM as described. Following 96 h of CM treatment, the cells were collected, counted, and plated in a 96-well plate at indicated concentrations. Ratios of treated bone marrow cells to splenocytes of 1:1, 1:2, 1:5 and 1:10 were plated such that the number of bone marrow cells per well equaled 2 × 10^5^ and the number of splenocytes were adjusted accordingly to meet the ratios stated. Splenocytes were obtained by physical disruption of a wildtype FVB spleen, followed by red blood cell lysis and resuspension as a single cell solution. Splenocytes were stained with CFSE (Invitrogen) at a concentration of 1 μM for 10 min at 37 °C. Untreated bone marrow cells that were not exposed to CM were used as a control. T cells were stimulated to proliferate using 1 μg ml^−1^ anti-mouse CD3 antibody (eBioscience). Following 72 h of stimulation, cells were collected and stained with anti-CD8 antibody to identify the effect on CD8^+^CFSE^+^ T-cell proliferation. Experiments were performed in triplicate.

### Subcutaneous injections and bioluminescence imaging

MSF cells were injected in to the flanks of FVB mice, two injections/mouse. Cells were injected at a concentration of 4 × 10^5^ cells/injection in 100 μl of a 1:1 solution (DMEM:growth factor-reduced matrigel (Corning). For co-injection experiments using PDSC5 and MK16-Ras tumour lines, the MSFs (4 × 10^5^/injection) were mixed before injection with either 1.5 × 10^5^ PDSC5 cells or 100 MK16-Ras cells per injection. Tumour growth for the MK16-Ras line was monitored using calipers. On day 26, MK16-Ras tumours were isolated for histological analysis. For the PDSC5 tumour line, bioluminescence imaging was used to monitor growth with images taken on days 2 and 9 following injection.

Bioluminescence imaging was performed using an IVIS Lumina (PerkinElmer; Living Image 3.2, 5 min exposure, bin8, FOV12.5 cm, f/stop1, open filter)^53^. Mice were injected intraperitoneally with D-luciferin (150 mg kg^−1^ in PBS; Gold Biotechnology) and imaged 10 min later under isoflurane anaesthesia (2% vaporised in O_2_).

*In vitro* live-cell bioluminescence imaging was performed on an IVIS 50 (PerkinElmer; Living Image 4.3, 5 min exposure, bin8, FOV12 cm, f/stop1, open filter). D-luciferin (150 mg ml^−1^; Gold Biotechnology) was added to black-walled plates 10 min before imaging. Total photon flux (photons sec^−1^) was measured from fixed regions of interest over the plate or tumours using Living Image 2.6.

### Tissue digestion and flow cytometry analysis

Isografts or dorsal skin (FASST and littermate) were isolated, minced, and transferred to a flask containing collagenase solution (2 mg ml^−1^ Collagenase A (Sigma), 1 mg ml^−1^ hyaluronidase (Sigma), 2 U ml^−1^ DNase I (Roche) in serum-free DMEM) and a magnetic stir bar. Flasks were placed on stir plates in a 37° incubator for 1 h. Solution was strained using a 70 μM cell strainer. Blocking was performed using F_c_ block (1:200, BD Pharmingen). Cell surface and intracellular staining was performed. Concentrations, clones and fluorophore conjugations can be found in Supplementary Table 1. For FoxP3 intracellular staining, Foxp3/Transcription Factor Staining Buffer Set (eBioscience) was used according to instructions (Protocol B). Cell staining was analysed using an LSRII flow cytometer (Becton Dixon) and FlowJo software (Tree Star).

### Neutralizing antibodies and dosing strategies

*In vivo* neutralizing antibodies and dosing strategies were as follows: IL-6 neutralizing antibody (clone: MP5-20F3, BioXcell) or control IgG (IgG1 isotype control, BioXcell) at a dose of 500 μg per mouse twice weekly starting 1 week before subcutaneous injection, Ly6G neutralizing antibody (clone: 1A8, BioXcell) or control IgG (IgG2a isotype control, BioXcell) at a dose of 1 mg per mouse for the first dose and 500 μg per mouse for each subsequent dose every 3 days, starting 9 days before subcutaneous cell injection, CD8 neutralizing antibody (clone: YTS 169.4, BioXcell) at a dose of 250 μg per mouse starting at day −2 and once every 7 days for the duration of the experiment. All antibodies were delivered by I.P. injection.

### IFNγ *ex vivo* activation assay

Isografts were disaggregated as described. For stimulation, PMA (phorbol 12-myristate 13-acetate) (Sigma), ionomycin (Sigma) and Brefeldin A (BioLegend) were added at the following concentrations: PMA 50 ng ml^−1^, ionomycin 0.709 μg ml^−1^, Brefeldin A 5 ug ml^−1^. Cells were plated onto 96-well plates using one well per mouse (2 isografts). The plate was incubated for 3 h at 37 °C then the contents of each well was transferred to a 1.5 ml eppendorf tube for staining. Initial cell surface staining was performed as described above (CD45-PE-Cy7, CD3-APC, CD8-Alexafluor 700) followed by fixation of the cells using 4% formaldehyde in PBS. After fixation, permeabilization buffer (BD Bioscience) was added, cells were spun down and resuspended in a wash of permeabilization buffer then spun down again before resuspension with antibodies against IFNγ or a control IgG (IFNγ-PE or IgG-PE). Intracellular staining was carried out for 1 h on ice in the dark. Cells were washed and transferred to FACS tubes for analysis using a BD LSRII cytometer (Becton Dixon) and FlowJo software (Tree Star). Additional information about antibodies can be found in Supplementary Table 1.

### Spatial and statistical analysis

Spatial analysis of GFP-positive senescent cells and CD45 positive immune cells was done by manually identifying cells (human samples) or by using automated cell detection techniques (mouse samples). Automated cell detection was performed using custom Matlab Software. Before processing, regions of the image other than the interstitial stroma were masked (outside the tissue, the epithelial layer, hair follicles, muscle tissue) were masked. In each image, the median distance from each immune cell to the nearest senescent cell was computed, providing an estimate of the observed nearest neighbour distance. The median was chosen over the mean to make this distance statistically less susceptible to outliers. To determine the distance between immune cells and senescent cells expected by chance, 100,000 rounds of Monte-Carlo sampling were performed by randomly placing the immune cells within the unmasked region of each image. For each round of sampling, the median distance between immune cells and the nearest senescent cell were recorded. The association score was computed by subtracting the observed distance from the mean sampling distance value and normalizing by the s.d. of the sampling distance distribution (dist_random—dist_observed)/(standard deviation). The score represents the number of s.d. the observed distance lies from the sampling distance in terms with positive values indicating association (observed closer than random) and negative values indicating repulsion (observed farther than random). A value of two corresponds to an observed median distance <97.5% of the random sampling median distances.

Statistical Analysis was done by calculating *P*-values using a standard Student's *t*-test with the exception of the following experiments: CD33 marker counts were modelled by a generalized mixed effects model with the negative binomial response distribution to investigate the fixed effect of senescence with the repeated measurements from various skin samples of each individual donor modelled as a random effect. For the PDSC5 shIL6 experiment, the fold growth of the log_2_ transformed BLI between day 9 and day 2 was analysed by a linear model with the effect of hairpin, the effect of senescence and the interaction between them. For the experiments on PDSC5 and MK16-Ras competent versus nude mice, log2 transformed BLI and Box-Cox transformed tumour size (with addition of 1 to accommodate the zeros) were analysed respectively. Note that several transformations (log, square root and Box-Cox) on the tumour size measurements were analysed and all gave similar conclusions on the senescence effect. The Box-Cox transformation (with the parameter estimated as 0.6747 using the R function ‘boxcox') resulted in the best goodness-of-fit and was reported. A linear model was separately fitted on the logged BLI and Box-Cox transformed tumor size incorporating the effect of mouse strain, the effect of senescence and their interaction. From the linear models, the effect of senescence within each hairpin or each strain of mice and the effect difference between hairpins or strains of mice were estimated and tested. All tests were two-sided and significance was defined at a 5% level. All analyses were conducted in R (version 3.1.1) (http://www.R-Project.org/).

### Data availability

All relevant data are available from the authors.

## Additional information

**Accession codes:** The RNA-seq data have been deposited in the GEO database (http://www.ncbi.nlm.nih.gov/geo/) under the accession code GSE78128.

**How to cite this article:** Ruhland, M. K. *et al*. Stromal senescence establishes an immunosuppressive microenvironment that drives tumorigenesis. *Nat. Commun.* 7:11762 doi: 10.1038/ncomms11762 (2016).

## Supplementary Material

Supplementary Information Supplementary Figures 1-7 and Supplementary Table 1
