# Supplementary material for: Stromal senescence establishes an immunosuppressive microenvironment that drives tumorigenesis
Source: Nat Commun. 2016 Jun 8;7:11762. doi: 10.1038/ncomms11762 (PMC4899869; doi:10.1038/ncomms11762)
Supplement: Supplementary Information — Supplementary Figures 1-7 and Supplementary Table 1 [file ncomms11762-s1.pdf]

SUPPLEMENTARY INFORMATION

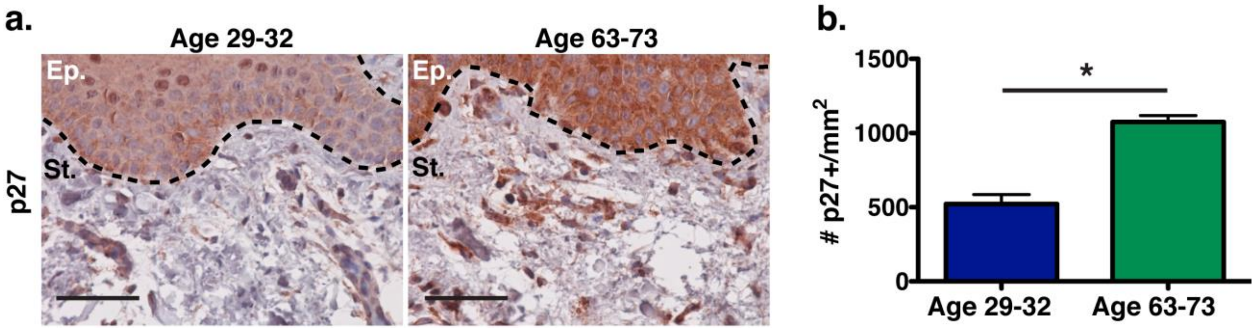

Supplementary Figure 1: Elevated level of p27^{Kip1} is found in aged human skin

a. Immunohistochemical staining for p27^{Kip1} in human skin samples grouped by age. The dashed line denotes the separation between the epithelium (Ep.) and stromal (St.) compartments. Scale bar = 50μm. Representative images. n = 4.

b. Quantification of p27^{Kip1}staining in the stroma shown in a. Analysis is grouped by age. * indicates p-value < 0.05 by Student's t-test. Data is presented as the mean of p27^{+} cells/mm^2 + SEM. Graph contains data from 4 donors per age group, multiple images (8-17) per donor.

a.

Gene Symbol	Gene Name	Rank in Gene List	Core Enrichment
IL6	interleukin 6	0	Yes
IL20RA	interleukin 20 receptor, alpha	76	Yes
SOCS3	suppressor of cytokine signaling 3	93	Yes
PIM1	pim-1 oncogene	103	Yes
CCND1	cyclin D1	154	Yes
IL28RA	interleukin 28 receptor, alpha	297	Yes
CSF2RB	colony stimulating factor 2 receptor, beta	302	Yes
IL15	interleukin 15	322	Yes
CCND2	cyclin D2	590	Yes
CLCF1	cardiotrophin-like cytokine factor 1	775	Yes
PIK3CB	phosphoinositide-3-kinase, catalytic, beta polypeptide	786	Yes
PTPN11	protein tyrosine phosphatase, non-receptor type 11	878	Yes
BCL2L1	BCL2-like 1	881	Yes
PIK3R5	phosphoinositide-3-kinase, regulatory subunit 5	897	Yes
OSMR	oncostatin M receptor	971	Yes

b.

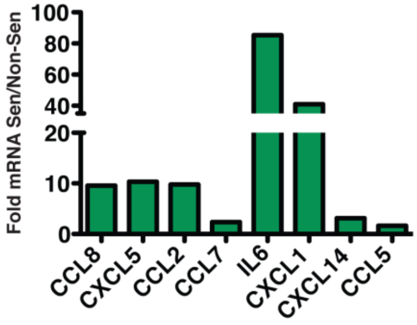

Supplementary Figure 2: RNA-Seq analysis from senescent versus non-senescent murine skin fibroblasts indicates an enhancement of factors regulated by the JAK-Stat pathway

a. Leading-edge pathway analysis of RNA-Seq data collected from senescence and non-senescence MSFs demonstrates increases in JAK-Stat pathway factors in senescence MSFs. Leading-edge analysis performed on GSEA results suggests the listed genes contribute to the core enrichment. n = 3

b. qRT-PCR validation of cytokines increased in senescent mouse skin fibroblasts detected by RNA-seq analysis in Fig. 1c. Data is presented as mean fold mRNA Sen/Non-Sen from triplicate wells. n = 3

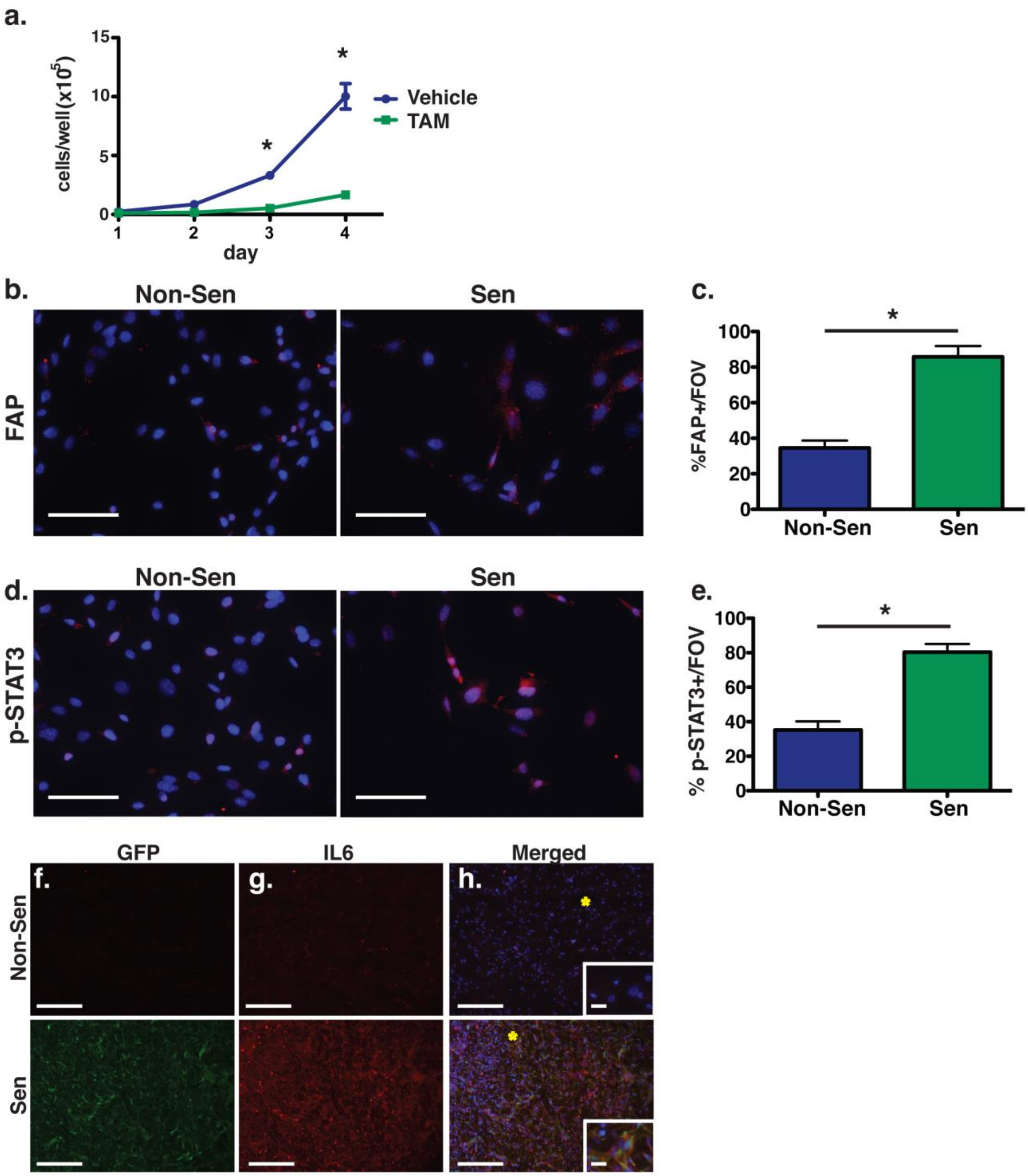

Supplementary Figure 3: Senescent MSFs persist *in vivo* and display increases in FAP, p-STAT3, and IL-6.

a. Growth curve of tamoxifen-treated (TAM) senescent mouse skin fibroblasts compared to vehicle-treated, non-senescent, control fibroblasts. Data is mean cells per well + and – SEM. * indicates p-value < 0.05 by ANOVA. Representative experiment. n = 3.

b. Immunofluorescence staining for fibroblast activation protein (FAP, red) in senescent (Sen) and non-senescent (Non-Sen) fibroblasts *in vitro.* Senescence was induced using 10μM tamoxifen. Nuclei are shown in blue (DAPI). Scale bar = 100μm. Representative images. n = 4-7.

c. Quantification of FAP immunofluorescence staining shown in b. Data is presented as the mean % of FAP+ cells + SEM per 20x field of view (FOV). * indicates p-value < 0.05 by Student's t-test. n = 4-7.

d. Immunofluorescence staining for phosphorylated STAT3 (p-STAT3, red) in senescent (Sen) and non-senescent (Non-Sen) fibroblasts *in vitro.* Senescence was induced using 10μM tamoxifen. Nuclei are shown in blue (DAPI). Scale bar = 100μm. Representative images. n = 15.

e. Quantification of p-STAT3 immunofluorescence staining shown in d. Data is presented as the mean % of FAP+ cells per field of view (FOV) + SEM. * indicates p-value < 0.05 by Student's t-test. n = 15.

f. GFP+, IL-6 expressing senescent fibroblasts persist in vivo. Immunofluorescence staining for GFP (green) and IL-6 (red) in senescence versus non-senescent isografts 11 days after injection. Nuclei are shown in blue (DAPI). Scale bar = 100μm. Representative images. n = 8.

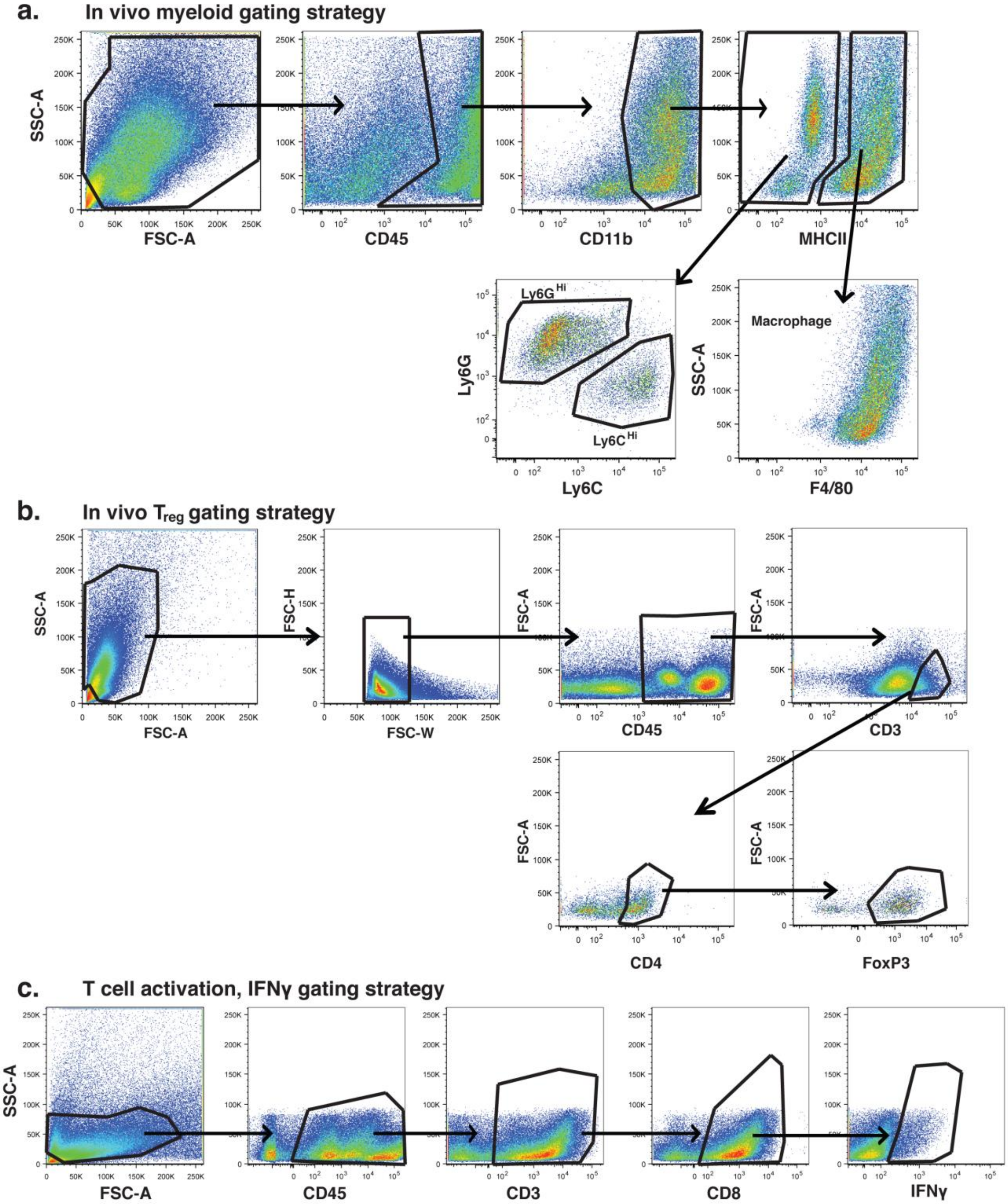

Supplementary Figure 4: Gating Strategies

a. Sequential gating strategy for myeloid-derived suppressor cells (Ly6G^{Hi} and Ly6C^{Hi}).

b. Sequential gating strategy for T regulatory (T_{reg}) cells based on CD4+FoxP3+ staining.

c. Sequential gating strategy for CD8+ T cell activation based on IFNγ+ staining.

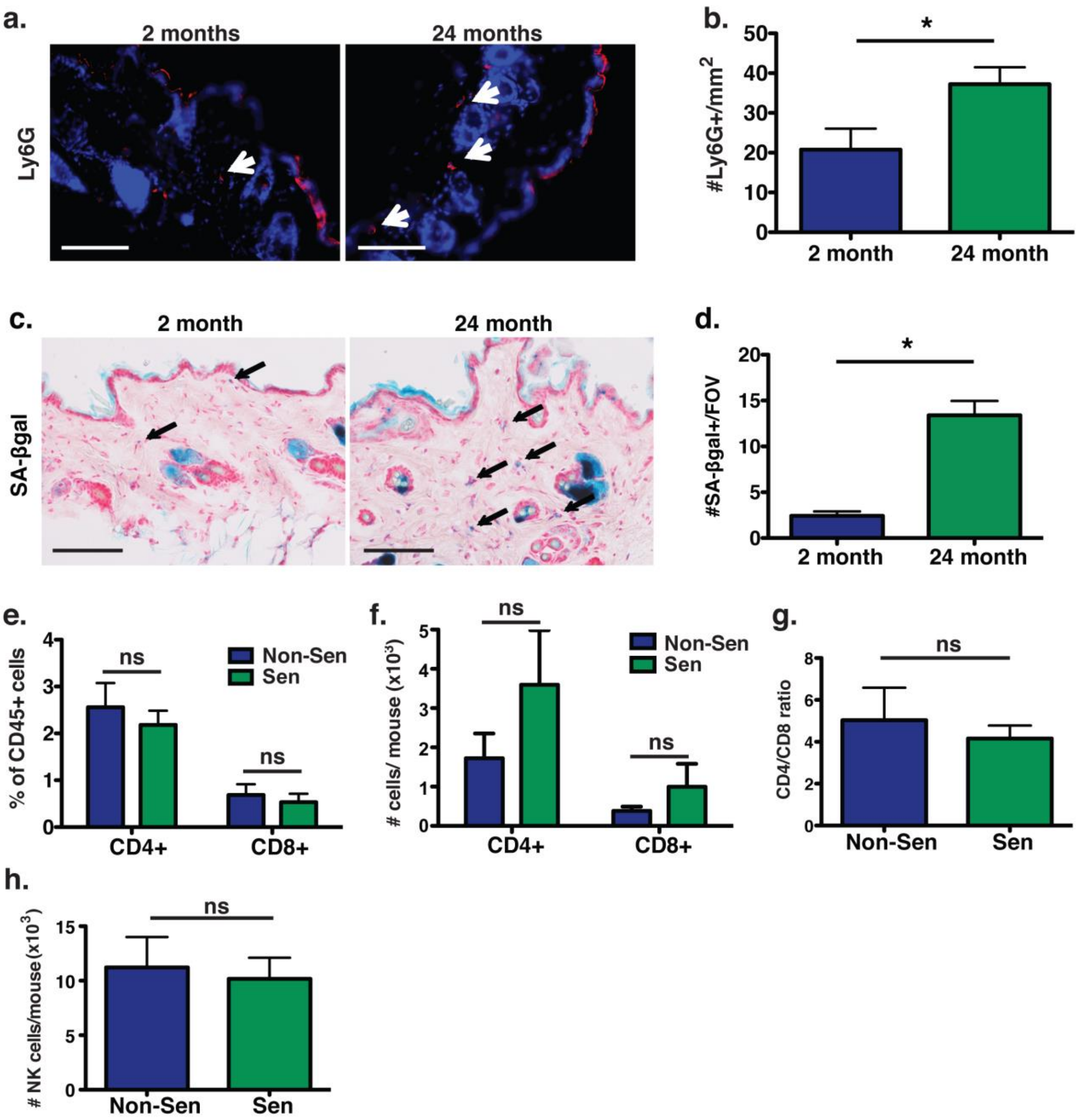

Supplementary Figure 5: Aged mice harbor increased SA-βgal $^{+}$ stromal cells and increased numbers of Ly6G^{+} cells

a. Immunofluorescence staining for Ly6G^{+} cells (red) in 2 month old (left) and 24 month old (right) mice. Nuclei are shown in blue (DAPI). Scale bar = 100μm. Representative images. n = 5-10

b. Quantification of Ly6G$^+$ cells staining shown in a. The stromal compartment was selected and ImageJ was used to calculate the area. Data are represented as mean Ly6G+cells/mm^2+ SEM. * indicates p-value < 0.05 by Student's t-test. n = 5-10

c. Senescence-associated β-galactosidase (SA-βgal) staining (blue) of 2 and 24 month old murine dorsal skin. Scale bar = 100μm. Representative images. n = 12-15

d. Quantification of SA-βgal stain in c. Data is presented as #SA-βgal$^+$ cells per field of view (FOV) + SEM. * indicates p-value < 0.05 by Student's t-test. n = 12-15

e. Quantification of CD4$^+$ or CD8$^+$ cells represented as % CD45$^+$ per mouse + SEM. ns is not significant by Student's t-test. n = 3-4

f. Quantification of CD4$^+$ or CD8$^+$ cells represented as the number of cells per mouse. ns is not significant by Student's t-test. Data is presented as the mean + SEM. n = 3-4

g. The ratio of CD4$^+$ to CD8$^+$ cells per mouse. ns is not significant by Student's t-test. Data is presented as the mean + SEM. n = 3-4

h. Quantification of NK cells represented as the # of cells/mouse. ns is not significant by Student's t-test. Data is presented as the mean + SEM. n = 3-5

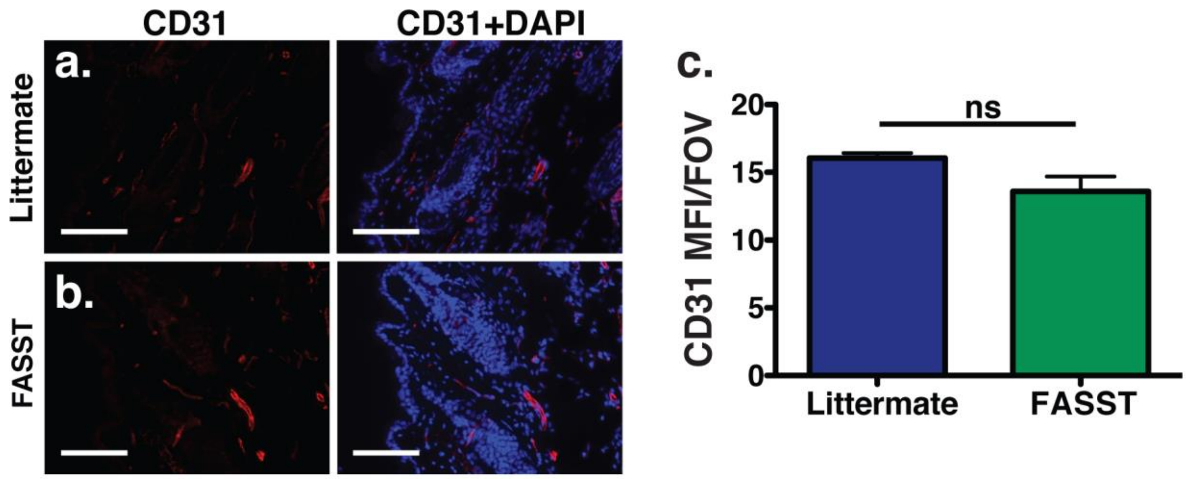

Supplementary Figure 6: Angiogenesis is not altered in FASST mice.

a. Immunofluorescent staining for $CD31^+$ cells (red) in littermate control mice. Nuclei are shown in blue (DAPI). Scale bar = 100μm. Representative images. n = 3

b. Immunofluorescent staining for $CD31^+$ cells (red) in FASST mice. Nuclei are shown in blue (DAPI). Scale bar = 100μm. Representative images. n = 3

c. Quantification of $CD31^+$ cells in littermate control versus FASST mice. Data are represented as mean fluorescence intensity (MFI)/field of view (FOV). ns is not significant by Student's t-test. Data is presented as the mean + SEM. n = 3

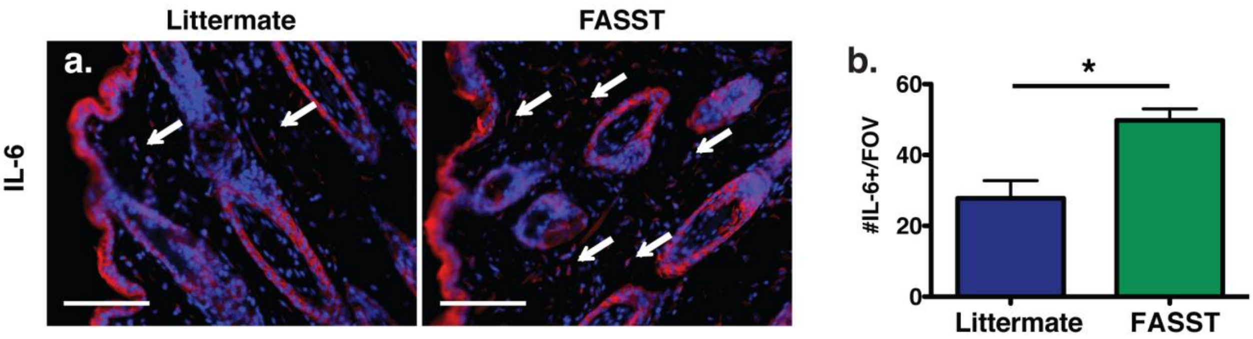

Supplementary Figure 7: IL-6 expressing senescent stromal cells are present in FASST mice.

a. Immunofluorescent staining for IL-6^{+} cells (red) in littermate control or FASST mice. Nuclei are shown in blue (DAPI). Scale bar = 100μm. Representative images. n = 4-5

b. Quantification of IL-6^{+} cells in littermate control versus FASST mice. Data are represented as cell number per field of view (FOV). * indicates p-value < 0.05 by Student's t-test. Data is presented as the mean + SEM. n = 4-5

Antibody name	Clone	Fluorophore	Assay Type	Concentration	Company	Cat. #.
**CONJUGATED (anti-mouse)**				(Dilution), final concentration		
**CD45**	30-F11	PE	IF	(1/200), 1μg ml^{-1}	BD Pharmingen	553081
**IgG control (CD45)**	A95-1	PE	IF	(1/200), 1μg ml^{-1}	BD Pharmingen	553989
**CD11b**	M1/70	PE	IF	(1/200), 1μg ml^{-1}	BD Pharmingen	557397
**IgG control (CD11b**	A95-1	PE	IF	(1/200), 1μg ml^{-1}	BD Pharmingen	553989
**Gr-1**	RB6-8C5	PE	IF	(1/400), 0.5μg ml^{-1}	eBioscience	12-5931-82
**IgG control (Gr-1)**	eB149/10H5	PE	IF	(1/400), 0.5μg ml^{-1}	eBioscience	12-4031-82
**CD45**	30-F11	PE-Cy7	FC	(1/1000), 0.2μg ml^{-1}	eBioscience	25-0451-82
**CD11b**	M1/70	Alexa Fluor 700	FC	(1/400), 0.5μg ml^{-1}	eBioscience	56-0112-82
**MHCII**	M5/114.15.2	eFluor 45	FC	(1/800), 0.25μg ml^{-1}	eBioscience	48-5321-82
**Ly6G**	1A8	PE	FC	(1/400), 0.5μg ml^{-1}	BioLegend	127607
**Ly6C**	HK1.4	APC	FC	(1/400), 0.5μg ml^{-1}	eBioscience	17-5932-82
**F4/80**	BM8	FITC	FC	(1/200), 2.5μg ml^{-1}	eBioscience	11-4801-82
**CD3**	145-2C11	APC	FC	(1/200), 1μg ml^{-1}	eBioscience	17-0031-82
**CD4**	H129.19	FITC	FC	(1/200), 2.5μg ml^{-1}	BD Pharmingen	553651
**FoxP3**	FJK-16s	PE-Cy5	FC	(1/25), 8μg ml^{-1}	eBioscience	15-5773-80
**CD8a**	53-6.7	Alexa Fluor 700	FC	(1/400), 0.5μg ml^{-1}	eBioscience	56-0081-80
**CD8a**	53-6.7	APC	FC	(1/800), 0.25μg ml^{-1}	BD Pharmingen	553035
**IFNγ**	XMG1.2	PE	FC	(1/200), 1μg ml^{-1}	BioLegend	505808
**IgG control (IFNγ)**	RTK2071	PE	FC	(1/200), 1μg ml^{-1}	BioLegend	400408
**CD49b**	DX5	APC	FC	(1/200), 1μg ml^{-1}	eBioscience	17-5971-81
**CD3**	145-2C11	FITC	FC	(1/200), 2.5μg ml^{-1}	BD Pharmingen	553062
**CD4**	GK1.5	PE	FC	(1/200), 1μg ml^{-1}	eBiosciene	12-0041-82

UNCONJUGATED (α-mouse unless specified)	Clone	Fluorophore	Assay Type	Concentration	Company	Cat. #.
**CD45**	30-F11	N/A	IHC	(1/100), 0.6μg ml^{-1}	BD Pharmingen	550539
**GFP**	chicken polyclonal	N/A	IF	(1/1000), 10μg ml^{-1}	Abcam	ab13970
**GFP secondary**	α-chicken	Alexa 488	IF	(1/1000), 1.5μg ml^{-1}	Jackson ImmunoResearch	103-545-155
**p16 (human)**	EPR 1473	N/A	IF	(1/100), 10μg ml^{-1}	Abcam	ab108349
**p16 (human) secondary**	α-rabbit	SA-Alexa 594	IF	(1/1000), 2μg ml^{-1}	Life Technologies	S-32356
**CD45 (human)**	HI30	N/A	IF	(1/100), 5μg ml^{-1}	eBioscience	14-0459-82
**CD45 (human) secondary**	α-mouse	Alexa 488	IF	(1/1000), 2μg ml^{-1}	Life Technologies	A-11001
**IL6**	goat polyclonal	N/A	IF	(1/50), 2μg ml^{-1}	Santa Cruz	sc-1265
**IL6 secondary**	α-goat	Alexa 594	IF	(1/1000), 2μg ml^{-1}	Life Technologies	A-11058
**CD31**	rabbit polyclonal	N/A	IF	(1/50), 0.4μg ml^{-1}	Abcam	ab28364
**CD31 secondary**	α-rabbit	Alexa 594	IF	(1/1000), 2μg ml^{-1}	Life Technologies	A-21207
**IL-6 (human)**	rabbit polyclonal	N/A	IF	(1/400), not purified	Novus Biologicals	NB600-1131
**IL-6 secondary (human)**	α-rabbit	Alexa 594	IF	(1/500), 4μg ml^{-1}	Life Technologies	A-21207
**FAPα**	rabbit polyclonal	N/A	IF	(1/200), 12.5μg ml^{-1}	Millipore	ABT11
**FAP secondary**	α-rabbit	Alexa 594	IF	(1/1000), 2μg ml^{-1}	Life Technologies	A-21207
**p-Stat3**	Rabbit polyclonal	N/A	IF	(1/50), 1.58μg ml^{-1}	Cell Signaling	9145

**p-Stat3 secondary**	α-rabbit	Alexa 594	IF	(1/500), 4μg ml^{-1}	Life Technologies	A21207

Supplementary Table 1: Antibody Information

All antibodies used for these studies are listed with their associated fluorophore if one was used, company, concentration (dilution), assay and catalog numbers. Abbreviations are as follows: R-phycoerythrin (PE); Fluorescein (FITC); Allophycocyanin (APC); Strepavidin (SA); Immunofluorescence (IF); Flow cytometry (FC); Immunohistochemistry (IHC)
